# AKT-mTORC1 reactivation is the dominant resistance driver for PI3Kβ/AKT inhibitors in PTEN-null breast cancer and can be overcome by combining with Mcl-1 inhibitors

**DOI:** 10.1038/s41388-022-02482-9

**Published:** 2022-10-14

**Authors:** Shanade Dunn, Cath Eberlein, Jason Yu, Albert Gris-Oliver, Swee Hoe Ong, Urs Yelland, Natalie Cureton, Anna Staniszewska, Robert McEwen, Millie Fox, James Pilling, Philip Hopcroft, Elizabeth A. Coker, Patricia Jaaks, Mathew J. Garnett, Beverley Isherwood, Violeta Serra, Barry R. Davies, Simon T. Barry, James T. Lynch, Kosuke Yusa

**Affiliations:** 1 Wellcome Sanger Institute, Cambridge, UK; 2 Bioscience, Early Oncology, AstraZeneca, Cambridge, UK; 3 Bioscience, Early Oncology, AstraZeneca, Alderley Park, UK; 4 Vall d’Hebron Institute of Oncology, Barcelona, Spain; 5 Discovery Sciences, AstraZeneca, Cambridge, UK; 6 Institute for Frontier Life and Medical Sciences, Kyoto University, Kyoto, Japan; 7 Present Address: Molecular Biology of Metabolism Lab, The Francis Crick Institute, London, UK

**Keywords:** Cancer therapeutic resistance, Target identification

## Abstract

The PI3K pathway is commonly activated in breast cancer, with PI3K-AKT pathway inhibitors used clinically. However, mechanisms that limit or enhance the therapeutic effects of PI3K-AKT inhibitors are poorly understood at a genome-wide level. Parallel CRISPR screens in 3 PTEN-null breast cancer cell lines identified genes mediating resistance to capivasertib (AKT inhibitor) and AZD8186 (PI3Kβ inhibitor). The dominant mechanism causing resistance is reactivated PI3K-AKT-mTOR signalling, but not other canonical signalling pathways. Deletion of *TSC1/2* conferred resistance to PI3Kβi and AKTi through mTORC1. However, deletion of *PIK3R2* and *INPPL1* drove specific PI3Kβi resistance through AKT. Conversely deletion of *PIK3CA*, *ERBB2*, *ERBB*3 increased PI3Kβi sensitivity while modulation of *RRAGC*, *LAMTOR1*, *LAMTOR4* increased AKTi sensitivity. Significantly, we found that *Mcl-1* loss enhanced response through rapid apoptosis induction with AKTi and PI3Kβi in both sensitive and drug resistant *TSC1/2* null cells. The combination effect was *BAK* but not *BAX* dependent. The Mcl-1i + PI3Kβ/AKTi combination was effective across a panel of breast cancer cell lines with *PIK3CA* and *PTEN* mutations, and delivered increased anti-tumor benefit in vivo. This study demonstrates that different resistance drivers to PI3Kβi and AKTi converge to reactivate PI3K-AKT or mTOR signalling and combined inhibition of Mcl-1 and PI3K-AKT has potential as a treatment strategy for PI3Kβi/AKTi sensitive and resistant breast tumours.

## Introduction

Breast cancer (BC) is a leading cause of cancer-related deaths in women worldwide. Despite significant improvements in survival rates, the treatment of advanced BC remains challenging, highlighting the need for new effective therapies. The PI3K/AKT/PTEN signaling pathway is frequently deregulated in advanced BC, as well as in other types of solid tumours. Deregulation of the PI3K/AKT/PTEN pathway occurs through multiple mechanisms including loss of the tumour suppressor PTEN along with activating mutations in *PIK3CA (*the catalytic subunit of PI3Kα), *AKT1* and *PIK3R1* (the regulatory subunit of the PI3K complex) [1–3]. Of these, the most frequent mutations are found in *PIK3CA* and *PTEN*. In preclinical BC models, activating mutations in *PIK3CA* correlate with sensitivity to PI3Kα and *AKT* inhibitors, while loss-of-function mutations in *PTEN* are associated with increased sensitivity to PI3Kβ and AKT inhibitors [4–8].

Inhibitors of the PI3K pathway have shown clinical activity in patients with advanced BC [9]. Alpelisib (PI3Kα inhibitor) in combination with fulvestrant (selective estrogen receptor degrader) is a treatment option for patients with estrogen-receptor positive (ER+)/HER2-negative advanced BC, previously exposed to endocrine therapy and with mutations in *PIK3CA*/PI3Kα [10]. Capivasertib, a potent selective inhibitor of all three AKT isoforms [4], in combination with fulvestrant shows clinical activity in patients with ER+/HER-negative BC, including those with *PIK3CA*/*AKT1/PTEN* mutant tumours [11]. Capivasertib and ipatasertib [12] (both AKT inhibitors) have both demonstrated clinical activity in combination with paclitaxel in triple-negative-breast cancer (TNBC) in Phase II clinical studies [13, 14], with more pronounced benefit in patients with *PIK3CA*/*AKT1*/*PTEN* mutant tumours. However a recent Phase III trial (IPATunity130) of ipatasertib in combination with paclitaxel was negative. Capivasertib is currently in Phase III development as a combination therapy for breast (ER + BC and TNBC) and prostate cancers, including patients with mutations in PI3K/AKT/PTEN pathway genes [11, 13, 15, 16]. Moreover capivasertib has also demonstrated single-agent clinical activity in patients with BC harbouring *AKT1* and *PIK3CA* mutant tumours and germ-line *PTEN* alterations [17–21].

While inhibitors of the PI3K/AKT/PTEN pathway have shown clinical activity in advanced BC, acquired and innate resistance to these agents limits their activity. Therefore, it is important to determine factors that limit, or enhance therapeutic response. PI3K pathway inhibitors are subject to both feedback-mediated reactivation of the pathway and resistance through secondary gene mutations. Acute feedback and PI3K/AKT pathway reactivation can occur through RTKs or switching signalling to alternate PI3K isoforms [22–24] or down-regulation of PTEN protein expression [25]. Additionally, co-activation of the ERK pathway or signalling through the mTORC1 complex can modify response to PI3K and AKT inhibitors [4, 26, 27]. Resistance to PI3Kα inhibitors in *PI3KCA* mutant BC has been studied both pre-clinically and clinically, and found to be associated with the loss of *PTEN*, resulting in activation of PI3Kβ [28, 29]. *PTEN* loss is also associated with clinical resistance to CDK4/6 and endocrine therapies in advanced BC [30].

Loss of *PTEN* is a common genetic alteration in advanced BC and is associated with poor prognosis [1, 2, 31, 32]. Almost half of TNBC tumours are deficient for PTEN expression [33]. Despite the frequency of *PTEN* loss in BC, little is known about the resistance mechanisms and pathway interactions that modify response to therapeutic targeting of the PI3K pathway in *PTEN*-deficient BC. To systematically identify and compare mechanisms of resistance to PI3K versus AKT pathway inhibition, we performed genome-scale CRISPR knockout (KO) screens in *PTEN*-deficient BC cells in the presence of AKT and PI3Kβ inhibitors. This revealed novel PI3K/AKT/PTEN pathway interactions and resistance drivers, and identified combination targets to improve therapeutic response and overcome resistance to PI3Kβ and AKT inhibitors.

## Results

### Genome-wide CRISPR-KO screens identify genes involved in PI3Kβ and AKT inhibitor resistance

**Fig. 1:**
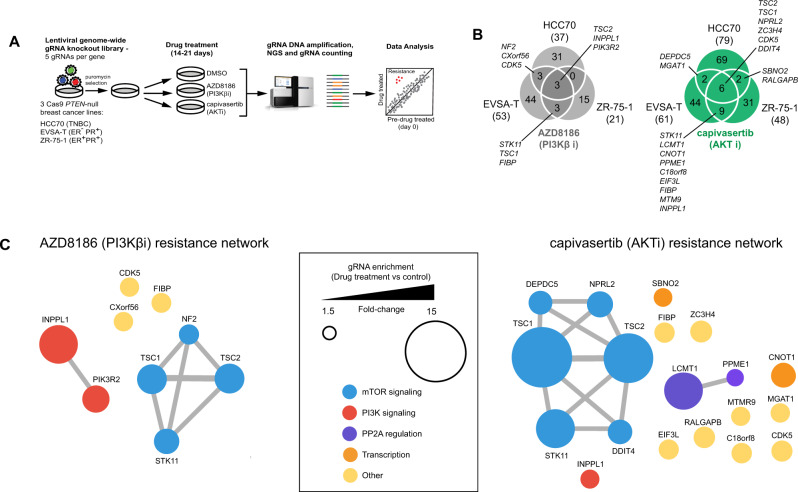
CRISPR screens identify genes involved in resistance to AKT and PI3Kβ inhibitors in *PTEN*-deficient breast cancer cell lines. **A** Outline of CRISPR screening approach used to identify gene knockouts that increase or decrease sensitivity to capivasertib and AZD8186. **B** Venn diagram showing overlap in AZD8186 and capivasertib resistance genes identified across the lines. **C** STRING network analysis of AZD8186 (left) and capivasertib (right) resistance genes identified in at least two cell lines (*n* = 9 for AZD8186 and *n* = 19 for capivasertib). Node size represents resistance effect caused by gene KO (mean gRNA FC for each gene in drug treated relative to DMSO control across three cell lines). Smallest and largest node represent mean gRNA FC of 1.5 and 15, respectively. Node colour represents pathway or associated gene function. Colour and size of node on STRING network were modified manually.

To elucidate mechanisms of resistance to PI3Kβ (AZD8186) and AKT (capivasertib) inhibitors in *PTEN*-deficient breast cancer, we performed genome-wide CRISPR knockout screens in three breast cancer (BC) cell lines with loss-of-function mutations in *PTEN* (Fig. 1A). The cell lines were HCC70 (TNBC), ZR-75-1 (ER+) and EVSA-T (PR+, ER−) and are highly sensitive to both compounds [4, 5].

The CRISPR-KO screens were performed using clonal Cas9-expressing HCC70, EVSA-T and ZR-75-1 cell lines with high gene knockout activity and similar pharmacological sensitivity to AZD8186 and capivasertib as the parental cell line (Supplementary Fig. 1A, B). Cells were transduced with a genome-wide CRISPR-KO lentiviral library [34] and then treated with either AZD8186 (PI3Kβ inhibitor), capivasertib (AKT inhibitor) or vehicle control (DMSO) for a total of 14–21 days depending on cell line growth rate (Fig. 1A). Depletion of gRNAs targeting genes involved in essential cellular processes in cells in the absence of drug treatment confirmed the CRISPR library worked effectively (e.g. *PIK3CB*, *AKT*) (Supplementary Fig. 2).

To identify genes where knockout confers PI3Kβ inhibitor (PI3Kβi) and AKT inhibitor (AKTi) resistance, gRNAs significantly enriched (false discovery rate (FDR) ≤ 0.01 and gRNA enrichment ≥1.5-fold change) in AZD8186 and capivasertib treated cells, compared to vehicle-control cells were identified using MAGeCK [35] **(**Fig. 1B, Tables S1, S2**)**. This revealed that only a small number genes confer PI3Kβ/AKT inhibitor resistance in multiple PTEN-null BC cell lines. For PI3Kβi resistance, gRNAs targeting 3 genes (*TSC2, INPPL1* and *PIK3R2*) were significantly enriched in all three AZD8186-treated cell lines, and a further 6 genes (*TSC1, STK11, NF2, CXorf56, CDK5* and *FIBP*) in two out of the three lines. For AKTi resistance, gRNAs targeting 6 genes (*TSC1, TSC2, NPRL2, ZC3H4, CDK5* and *DDIT4*) were significantly enriched in all three capivasertib-treated cell lines and 13 genes (*DEPDC5, STK11, LCMT1, PPME1, INPPL1, CNOT1, SBNO2, FIBP, MTMR9, MGAT1, C18orf8*, *EIF3L and RALGAPB*) in two out of the three lines (Fig. 1B). The genes (9 genes for AZD8186 and 19 genes for capivasertib) were analysed using STRING network mapping (Fig. 1C). This revealed that PI3Kβ and AKT inhibitor resistance is associated with a single dominant network of mTORC1 signaling genes (including *TSC1, TSC2* and *STK11*). The TSC1-TSC2 complex is a critical negative regulator of mTORC1 signaling [36]. Given that TSC1 and TSC2 were identified as the strongest drivers of AKT inhibitor resistance in our CRISPR screen, and the TSC1-2 complex has previously been associated with PI3Kα inhibitor resistance [37, 38], the regulation of mTORC1 signaling by this complex appears to be a dominant driver of resistance to PI3K/AKT inhibition. Resistance to PI3Kβ inhibition was conferred by two PI3K signaling related genes *INPPL1* (encoding SHIP2 protein) and *PIK3R2* (encoding P85β protein). *INPPL1* was the strongest driver of PI3Kβ inhibitor resistance in our screen. *INPPL1* and *PIK3R2* are known regulators of PI3K/AKT signaling, but their effect on downstream signaling in BC remains unclear and neither gene has been linked to resistance to PI3K/AKT inhibitors previously. Beyond the PI3K/AKT/mTOR pathway, genes involved in PP2A regulation (*LCMT1*, *PPME1*), transcription regulation (*CNOT1*, *SBNO2*) and several uncharacterised genes (including *CXorf56*, *FIBP*, *C18orf8* and *ZC3H4*) were identified, which may reflect novel resistance mechanisms; although resistance caused by these genes was much weaker than the PI3K pathway related genes (such as *INPPL1* and *TSC2*). In summary, resistance to PI3Kβ/AKT inhibition in PTEN-null BC cells is conferred by loss of a specific genes in the PI3K/AKT/mTOR pathway (including *INPPL1*, *PIK3R2* and *TSC1/2*), rather than genes associated with alterative signaling pathways.

### Resistance to PI3K/AKT inhibition is mediated by specific genes in the PI3K-AKT-mTORC1 pathway reactivating downstream signaling

**Fig. 2:**
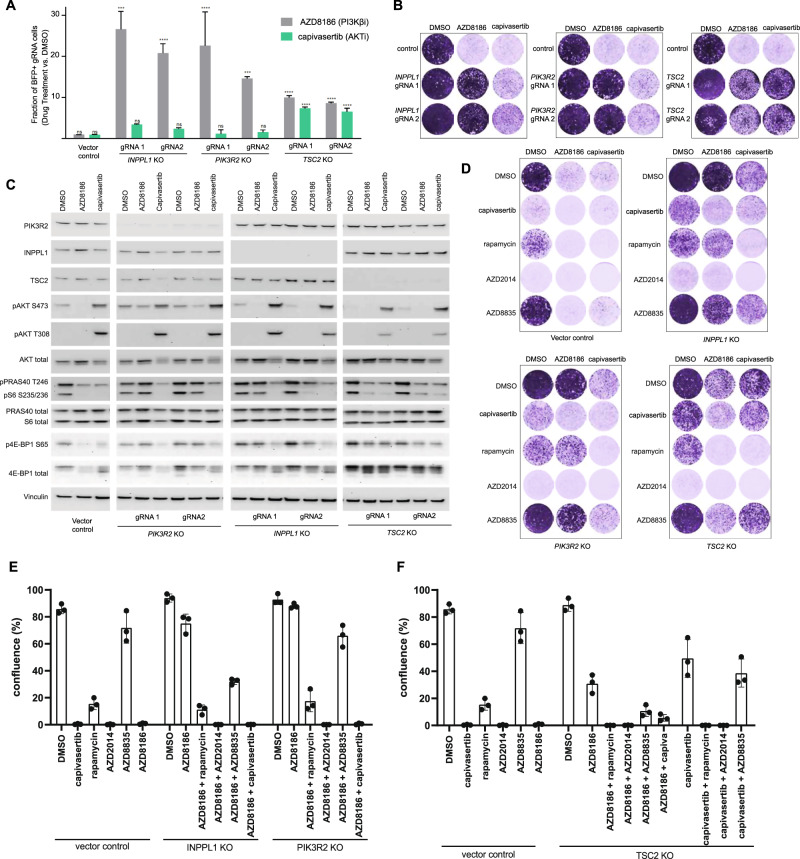
Loss of INPPL1, PIK3R2 and TSC2 cause drug resistance through re-activation of PI3K-AKT-mTOR pathway signaling. **A** Bar chart of results from the competitive proliferation assay for *INPPL1*, *TSC2* and *PIK3R2* KO and vector control cells. Data are mean ± SD; *n* = 3. Statistical significance was determined using Dunnett’s multiple comparisons test (****p < 0.0001, ***p < 0.001). **B** Crystal violet staining of EVSA-T *INPPL1*, *PIK3R2* and *TSC2* KO cells treated with DMSO, 250 nM AZD8186 or 1 μM capivasertib for 10 days. Data are representative of three independent experiments. **C** Western blot analysis of PI3K pathway effectors (pAKT, pPRAS40, pS6, p4E-BP1) in EVSA-T *INPPL1*, *TSC2* and *PIK3R2* KO cells treated with AZD8186, capivasertib or DMSO control for 4 h. Data are representative of three independent experiments. **D** Control and KO cells as indicated were treated with AZD8186 and capivasertib alone and with the addition of 1μM capivasertib, 100 nM Rapamycin, 500 nM AZD2014 and 500 nM AZD8835 combinations. Data are representative of three independent experiments and two independent gRNAs. **E**, **F** Plates from (**D**) were quantified using ImageJ software and % confluency of each well calculated. Data are mean of 3 independent experiments ± SD. All images shown are from the same blots at the same exposures, panels for each marker have been separated to aid visualisation, but images for each marker are comparable between KO cell lines and treatments.

The screening data revealed that specific genes (*TSC2, INPPL1, PIK3R2*) within the PI3K-AKT-mTOR pathway are the dominant mediators of PI3Kβ/AKT inhibitor resistance (Fig. 1). To confirm these findings the ability of *INPPL1, PIK3R2* and *TSC2*-deleted PTEN-null BC cells to proliferate in the presence of AZD8186 (PI3Kβi) or capivasertib (AKTi) was tested in competitive and clonogenic growth assays. Consistent with our screening data, deletion of *INPPL1* and *PIK3R2* conferred strong resistance only to PI3Kβ inhibition whereas deletion of *TSC2* conferred similar levels of resistance to AKT and PI3Kβ inhibition in both assays (Fig. 2A, B and Supplementary Fig. 3A–C).

We hypothesised that deletion of *INPPL1*, *PIK3R2* and *TSC2* confers resistance through re-activation of downstream PI3K pathway signaling following PI3K and AKT inhibition. AKT (p-PRAS40) and mTORC1 signaling (p-S6 and p-4EBP1) were suppressed following AZD8186 and capivasertib treatment in control cells. In *TSC2*-deleted cells (resistant to PI3K and AKT inhibition) AZD8186 and capivasertib treatment reduced p-PRAS40 but did not fully inhibit p-S6 and p-4EBP1 (downstream targets of mTORC1), indicating sustained mTORC1 signaling, or downstream mRNA translation, following *TSC2* loss causes PI3Kβi and AKTi resistance via an AKT-independent mechanism. In contrast, in *INPPL1 and PIK3R2* deleted cells, which are resistant to PI3Kβ inhibition, AZD8186 did not suppress p-PRAS40, p-S6 and p-4EBP1 but capivasertib was still effective, indicating loss of *INPPL1* and *PIK3R2* causes resistance to PI3Kβ inhibition through sustained AKT signaling (Fig. 2C and Supplementary Fig. 3). To explore this further *TSC2*, *INPPL1* and *PIK3R2*-deleted cells were treated with AKT (capivasertib), PI3Kα (AZD8835), mTORC1 (rapamycin) and mTORC1/2 (AZD2014) inhibitors and cell proliferation measured in the presence and absence of AZD8186 and capivasertib treatment. In *INPPL1 and PIK3R2*-deleted cells resistance to PI3Kβi was strongly reversed by capivasertib (AKT inhibition), AZD2014 (mTORC1/2 inhibition), partially reversed by rapamycin (mTORC1 inhibitor) while AZD8835 (PI3Kα/δ inhibitor) was less effective suggesting loss of *INPPL1* and PIK3R2 predominately causes PI3Kβi resistance through re-engagement of AKT signaling (Fig. 2D, E and Supplementary Fig. 3). PI3Kβi and AKTi resistance in *TSC2-*deleted cells was completely reversed by mTORC1 inhibition (rapamycin) or mTORC1/2 inhibition (AZD2014) (Fig. 2D, F and Supplementary Fig. 3). Interestingly increasing pathway inhibition by combining AZD8186 and AZD8835 (PI3Kα/δ inhibitor) or capivasertib gave marked growth inhibition, although residual resistance cells remained, but AZD8835 was not able to reverse capivasertib resistance. In summary, our data confirmed deletion of *TSC2* in PTEN null cells causes persistent cell growth or resistance to PI3Kβ/AKT inhibition through reactivation of mTORC1 signaling with possible increased signal through the PI3Kβ and AKT pathway. Loss of *INPPL1* and *PIK3R2* causes resistance to PI3Kβ inhibition resistance through reactivation of AKT signaling that is prevented by capivasertib and mTORC1/2 (AZD2014) treatment. This suggests that activation at different points on the PI3K-mTOR-AKT pathway drive resistance or persistent cell growth, which can be overcome by targeting specific points in the pathway.

### Mcl-1 inhibition sensitizes PTEN-null breast cancer cells to AKT and PI3Kβ inhibition

**Fig. 3:**
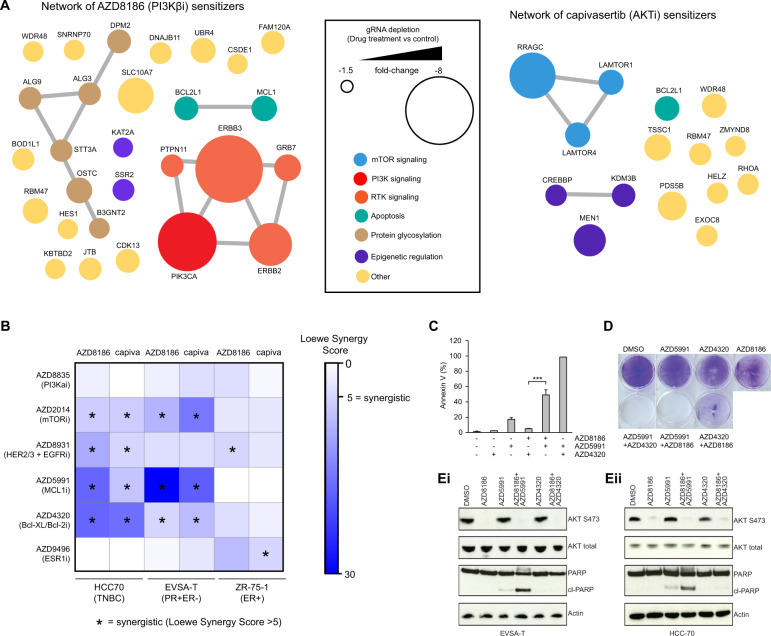
Identification and validation of Mcl-1 as a AKT and PI3Kβ inhibitor sensitizer. **A** STRING network analysis of interactions between AZD8186 (left) and capivasertib (right) sensitisation genes identified in at least two lines (*n* = 28 for AZD8186 and *n* = 15 for capivasertib). Node size corresponds to drug sensitization effect (mean gRNA FC for each gene across cell lines). Node colour represents pathway or function associated with gene. Smallest and largest node represents a FC of −1.5 and −8, respectively. Colour and size of node on STRING network were modified manually. **B** Heatmap representing synergy scores from a 5-day combination proliferation assay in EVSA-T, HCC70 and ZR-75-1 cells. Synergy scores determined using the Loewe additivity model. **C** Apoptosis induction analysed by Annexin V staining after 2-hour treatment. Data are mean ± SD; *n* = 3. **D** Crystal violet staining after 4-day treatment. **E** Western blot analysis in EVSA-T (**Ei**) and HCC70 (**Eii**) treated with AZD5991, AZD8186, AZD4320 or in combination. Data shown is representative of two independent experiments. For EVSA-T, 50 nM AZD5991, 500 nM AZD4320, 250 nM AZD8186 were used. For HCC70, 200 nM AZD5991, 100 nM AZD8186 and 500 nM AZD4320 were used.

While combining different agents targeting PI3K-mTOR-AKT signaling is an attractive strategy to maximise efficacy or address resistance in PTEN null cells, combinations can lead to increased toxicity, or agents targeting appropriate points on the pathway may not be currently in clinical development. To identify new drug combinations to enhance the efficacy of AKT and PI3Kβ inhibitors, screening data was analysed for genes that when deleted increase the sensitivity of PTEN-null BC cells to capivasertib and AZD8186. Sensitizer genes were identified as gRNAs significantly depleted (FDR ≤ 0.2 and gRNA enrichment ≤ −1.5 fold-change) in AZD8186 and capivasertib treated cells, compared to vehicle-control cells using MAGeCK (Fig. 3). Consistent with the resistance genes identified (Fig. 1), sensitivity to PI3Kβ inhibition was strongly influenced by modulation of PI3K-AKT signalling. STRING network mapping of these genes revealed that the dominant enhancers of PI3Kβ inhibitor response involved genes in RTK-signalling (*ERBB2, ERBB3, GRB7* and *PTPN11*) and PI3Kα signalling *(PIK3CA*), consistent with previous reports [24]. In contrast, AKT inhibitor activity was enhanced by loss of *RRAGC*, *LAMTOR1* and *LAMTOR4*, which are critical mediators of mTORC1 signaling. These findings suggest that in addition to mediating acquired AKT inhibitor resistance (Figs. 1, 2), mTORC1 signalling also limits the primary therapeutic response to AKT inhibition. Beyond the PI3K/AKT pathway, the anti-apoptotic genes *BCL2L1* (encoding Bcl-XL) and *MCL1* (encoding Mcl-1) stood out as strong sensitizers for AZD8186/capivasertib inhibitors. As *MCL1* loss inhibits the growth of EVSA-T cells in the absence of capivasertib, it was not a significant sensitizer for capivasertib in this line. However manual inspection of the gRNA abundance data from the CRISPR screen confirmed *Mcl-1* gRNA levels were further depleted in the presence of capivasertib (data not shown), indicating loss of Mcl-1 sensitizes to AKT inhibitor treatment.

To evaluate drug combination partners for PI3K/AKT inhibitors, drugs approved or in clinical development targeting the strongest sensitizers (*ERBB3*, *ERBB2*, *PIK3CA*, *BCL2L1* and *MCL1)* were combined with AZD8186 and capivasertib. HCC70, EVSA-T and ZR75-1 cells were treated with AZD8186 and capivasertib in combination with an inhibitor of either PI3Kα/δ (AZD8835), mTORC1/2 (AZD2014), Her2/3 and EGFR (AZD8931), Mcl-1 (AZD5991) or Bcl-XL/Bcl-2 (AZD4320) for five days and a Loewe dose-additivity synergy score was established for each combination (Fig. 3B). Combinations with inhibitors of anti-apoptotic agents AZD5991 (Mcl-1 inhibitor) and AZD4320 (Bcl-XL/Bcl-2 inhibitor) demonstrated the strongest benefit. Additional studies comparing the response of AZD5991 and AZD4320 when combined with AZD8186 revealed that Mcl-1 inhibition induced a greater degree of apoptosis and a greater reduction in cell number in EVSA-T and HCC70 cells compared to Bcl-XL inhibition (Fig. 3C–E). In summary, we have identified Mcl-1 as a new combination partner for PI3K and AKT inhibitors in PTEN-deficient breast cancer.

### Mcl-1-PI3Kβ/AKT inhibitor combination rapidly induces apoptosis in PTEN-deficient breast cancer cells

**Fig. 4:**
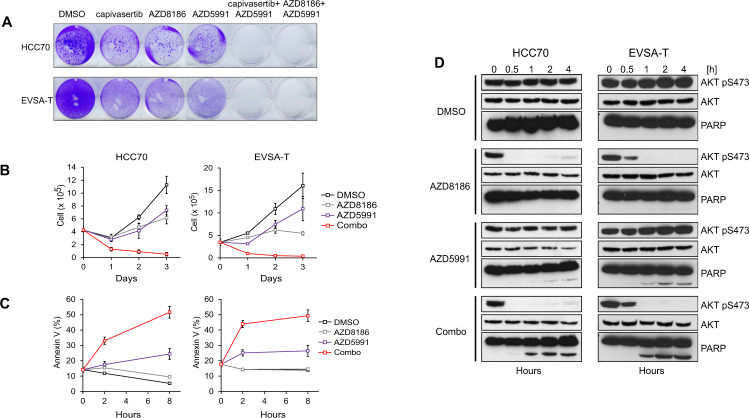
Mcl-1 and AKT/PI3Kβ inhibitor combination rapidly induces apoptosis in *PTEN*-deficient breast cancer cells. **A** Crystal violet staining in EVSA-T and HCC70 cells treated with the indicated drugs. **B** Proliferation of EVSA-T and HCC70 cells treated with AZD5991, AZD8186 or in combination for 3 days. Cells were counted every 24 h. Data are mean ± SD; *n* = 3. **C**, **D** Measurement of apoptosis induction in EVSA-T and HCC70 cells treated with time course of AZD5991, AZD8186 or combination for the times indicated and measured by Annexin V staining (**C**), or western blot analysis of cleaved PARP and PI3K pathway effector (pAKT S473) (**D**). Data are mean ± SD; *n* = 3. Data shown in **D** are representative of two independent experiments.

Mcl-1 prevents induction of apoptosis in many cancer types. We determined whether AZD8186 and capivasertib combines with the Mcl-1 selective inhibitor AZD5991 to reduce cell viability in PTEN-deficient BC cells. Crystal violet staining in EVSA-T and HCC-70 cells demonstrated that the combinations of AZD5991 with capivasertib/AZD8186 induced a striking reduction in cell viability compared with either drug alone (Fig. 4A). The combination of AZD5991 and AZD8186 led to a significant decrease in cell number below the starting cell density within 24 h consistent with decrease in cell survival and the induction of apoptosis (Fig. 4B). Single agent treatment of AZD5991 or AZD8186 had little effect on cell number at this timepoint. Next, the kinetics of the pro-apoptotic effect of AZD8186 and capivasertib combined with Mcl-1 inhibition was assessed. We found that the proportion of Annexin V positive cells (apoptotic cells) increased over 8 h, with increased apoptosis detected as early as 2 h following combination treatment (Fig. 4D). Although single treatment with AZD5991 caused a modest increase in apoptotic cells, the combination with AZD8186 induced a significantly higher proportion of apoptotic cells compared to single treatment in both EVSA-T and HCC70 cells. In agreement with the combination rapidly inducing apoptosis, western blot analysis confirmed that cleaved PARP was apparent following 1 h of combination treatment (Fig. 4D). This demonstrates that PI3Kβ/AKT inhibition in combination with Mcl-1 inhibition rapidly induces cell death in *PTEN*-deficient BC cells relative to single agent treatment. At high concentrations, AZD5991 was able to induce apoptosis as a monotherapy (data not shown). At lower AZD5991 doses, the combination with AZD8186 or capivasertib increased cell death, consistent with the hypothesis that PI3Kβ/AKT inhibition primes cells to Mcl-1 inhibition induced cell death.

### Mcl-1-PI3Kβ/AKT inhibitor combination effectively kills breast cancer cells resistant to PI3K/AKT inhibition

**Fig. 5:**
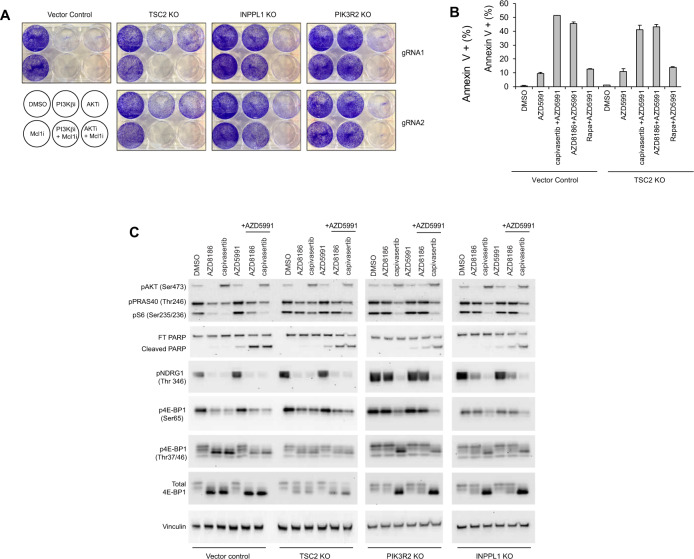
Mcl-1 and AKT/PI3Kβ inhibitor combination effectively kills PTEN-deficient breast cancer cells resistant to AKT/PI3Kβ inhibitors. **A** Crystal violet staining in EVSA-T *TSC2*, *INPPL1* and *PIK3R2* KO cells treated with the indicated drugs. **B** Annexin-V staining in EVSA-T *TSC2* knockout and control cells treated with the indicated drugs for 2 h. **C** Western blot analysis of PI3K pathway effectors (pAKT, pPRAS40, pS6,p4E-BP1), pNDRG1 and PARP in EVSA-T *INPPL1*, *TSC2* and *PIK3R2* KO cells treated with the indicated drugs for 4 h. Data are representative of three independent experiments.

Deletion of *TSC2*, *INPPL1* or *PIK3R2* mediates resistance to PI3K/AKT inhibition through re-activation of downstream PI3K pathway signaling (Figs. 1, 2). We next tested whether Mcl-1 inhibition using AZD5991 is effective at overcoming PI3Ki and AKTi inhibitor resistance in *PTEN*-null breast cancer cells lacking *PI3KR2*, *INPPL1* and *TSC2*. In *PIK3R2* and *INPPL1* KO cells (where AZD8186 resistance in caused by sustained AKT signaling) the combination of AZD8186 and AZD5991 was ineffective, but the combination of capivasertib and AZD5991 was still effective (Fig. 5A, C and Supplementary Fig. 4A, B). In *TSC2* KO cells which are resistant to AZD8186 and capivasertib through re-activation of mTOR signaling, both combinations (AZD5991 + capivasertib, AZD5991 + AZD8186) strongly reduced cell proliferation and induced cell death (Fig. 5A–C and Supplementary Fig. 4C), compared to each drug alone. The combination of AZD5991 and rapamycin had very minimal effects on the proportion of Annexin V positive KO and WT cells, demonstrating that Mcl-1 inhibition does not enhance the efficacy of mTOR inhibitors (Fig. 5B). Deletion of *TSC2*, *INPPL1* or *PIK3R2* did not change expression of pro- or anti-apoptopic BCL2 family members suggesting the effects are through priming of the apoptopic response through regulation of alternative apoptopic mechanisms (Supplementary Fig. 4D). Collectively our data demonstrates that combined inhibition of Mcl-1 and AKT/PI3Kβ is effective at killing breast cancer cells resistant to PI3Ki and AKTi in which resistance is caused by sustained mTORC1 signaling.

### Combined inhibition of PI3K/AKT and Mcl-1 is efficacious in *PTEN*-null cell line and patient-derived tumour xenografts

**Fig. 6:**
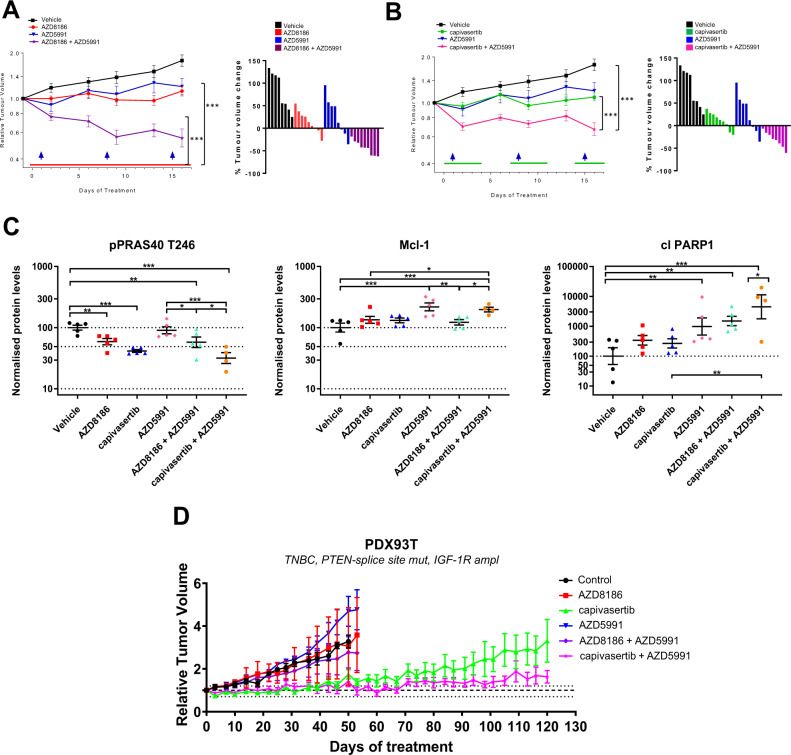
Mcl-1 and AKT/PI3Kβ inhibitor combination induces regression in *PTEN*-deficient breast cancer xenograft models. **A**, **B** HCC70 xenografts were treated with AZD5991 (60 mg/kg QW), AZD8186 (66.6 mg/kg QD), capivasertib (130 mg/kg BID) and in combination (*n* = 9 in each group). Geometric mean of the relative tumour volume and SEM are shown. Dosing schedule represented by the arrows and lines on the plots. **C** The analysis of pharmacodynamics markers. HCC70 tumour xenografts were treated for two days with AZD5991, AZD8186, capivasertib and in combination and analysed for the indicated markers. Plots represent geometric mean ± SEM biomarker signal. *p < 0.05, **p < 0.01, ***p < 0.001. *n* = 3–5 animals. **D** PDX93T tumours were treated with vehicle control (*n* = 4), AZD5991 (*n* = 5), AZD8186 *n* = 2), capivasertib (*n* = 8), AZD8186 and AZD5991 (*n* = 4), capivasertib and AZD5991 (*n* = 7) as indicated. Geometric mean of the relative tumour volume and SEM are shown. QW = once weekly; QD = once daily; BID = twice a day.

To investigate whether AZD8186 or capivasertib combined with AZD5991 enhances anti-tumor activity in vivo, both combinations were tested in mice bearing *PTEN*-null HCC70 tumor xenografts. Monotherapy treatment with AZD8186, capivasertib or AZD5991 reduced tumour growth relative to vehicle controls (Fig. 6A, B). In contrast, combining AZD8186 or capivasertib with AZD5991 resulted in significant tumour regression compared to the monotherapy. AZD5991 in combination with AZD8186 or capivasertib was well tolerated with minimal effects on body weight in both tumour models (Supplementary Fig. 5). Mechanistic biomarker analysis showed reduction in p-PRAS40, in all groups treated with capivasertib or AZD8186 alone or in combination (Fig. 6C and Supplementary Fig. 6). Mcl-1 levels were increased due to protein stabilization in the AZD5991 monotherapy and capivasertib+AZD5991 group confirming target engagement, however this increase was not apparent in the AZD8186 combination treated groups (Fig. 6C). Why Mcl-1 was not increased in the AZD8186 combination group versus the capivasertib treated combination group is unclear. Consistent with the in vitro observation (Figs. 3, 4), a significant increase in cleaved PARP was detected in AZD5991 monotherapy treated tumors, which was further increased when combined with AZD8186 or capivasertib, consistent with enhanced induction of apoptosis in vivo (Fig. 6C).

To expand on the efficacy observed in the HCC70 xenograft, the combination was further explored in a *PTEN*-deficient TNBC PDX model. This model has an amplification of *IGFR*. Based on published data [24], amplification of *IGFR* would render the tumor resistant to the effects of AZD8186 (both in terms of efficacy and AKT modulation), but retain sensitivity to capivasertib. As predicted AZD8186 and the combination of AZD8186 and AZD5991 were ineffective in this model (Fig. 6D). Capivasertib monotherapy showed initial anti-tumor efficacy in all 7 tumours, but three out of the 7 tumours eventually regrew on treatment (Fig. 6D). In contrast, the combination of capivasertib and AZD5991 resulted in durable tumor suppression and no tumors showed regrowth for the duration of this experiment (Fig. 6D and Supplementary Fig. 6). Collectively, our results demonstrate that the combination of targeting the PI3K pathway through inhibition of PI3Kβ or AKT and activating the pro-apoptotic pathway through inhibition of Mcl-1 is able to deliver greater anti-tumour benefit in vivo at tolerable doses in *PTEN*-deficient breast tumors.

### Mcl-1-PI3Kβ/AKT inhibitor combination is active across a panel of PI3K/AKT inhibitor resistant and sensitive breast cancer cell lines

**Fig. 7:**
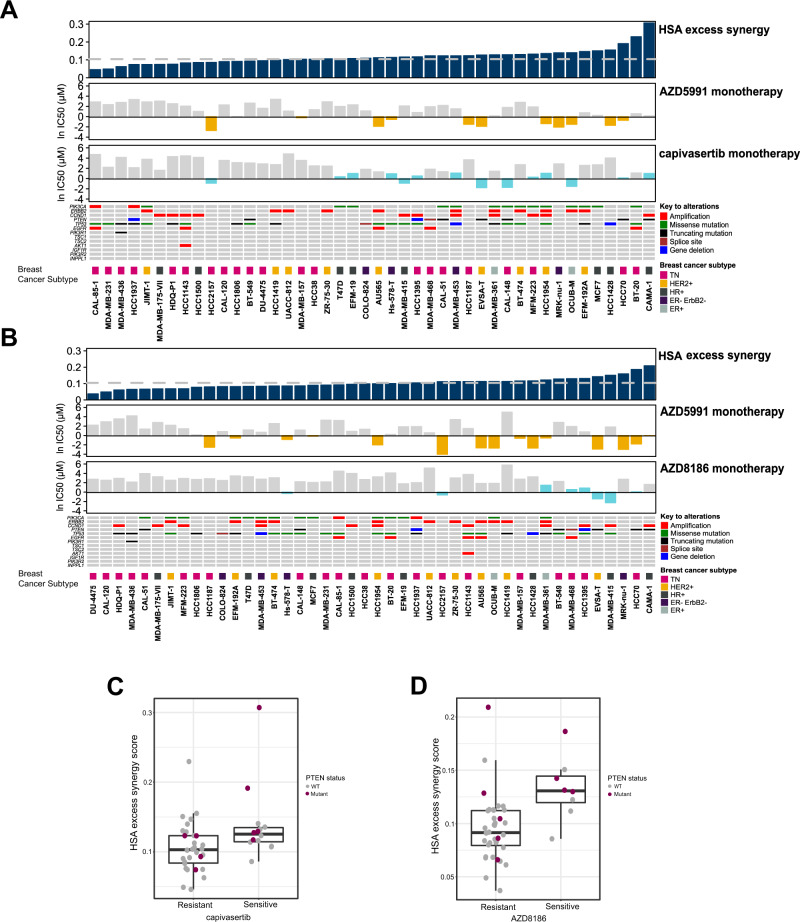
Mcl-1 and AKT/PI3Kβ inhibitor combination activity in a panel of 44 resistant and sensitive breast cancer cell lines. Plots showing the output of (**A**) AZD5991 + capivasertib and (**B**) AZD5991 + AZD8186 combination screens across a panel of BC cell lines in a 3-day proliferation assay. HSA excess synergy scores determined using the HSA synergy model. Monotherapy IC50 values are plotted as ln IC50 values, with a sensitivity cut-off of 1 µM AZD5991, 3 µM capivasertib and 3 µM AZD8186, indicated by the orange and blue bars, respectively. **C** Boxplots showing the HSA synergy scores from the AZD5991-capivasertib combination screen grouped by capivasertib sensitive (*n* = 14) and resistant (*n* = 30) BC cell lines. Significance was determined using a Wilcoxon signed-rank test. Purple dots represent *PTEN*-mutant cell lines. **D** HSA synergy scores from the AZD5991-AZD8186 combination screen grouped by AZD8186 sensitive (*n* = 8) and resistant (*n* = 36) BC cell lines.

To determine how broadly AZD5991 (Mcl-1 inhibition) combines with AZD8186 or capivasertib and to identify determinants of combination sensitivity, both combinations were assessed in a panel of 44 breast cancer cell lines (Fig. 7) [39]. The combination of capivasertib and AZD5991 gave synergistic inhibition of cell growth or survival as measured by a highest single agent (HSA) synergy score of >0.1 in 29/44 cell lines (Fig. 7A), and in 21/44 of cell lines with AZD8186 combined with AZD5991 (Fig. 7B). Capivasertib monotherapy activity was observed across a number of cell lines that harbour PI3K pathway mutations including PI3Kα, PTEN and ERBB2 amplification, although activity was also seen in non-pathway mutant cells. AZD8186 monotherapy activity was predominantly observed in PTEN altered cell lines, but again PTEN WT cells were also sensitive. Interestingly, combination activity was observed in cell lines that were not sensitive to capivasertib/AZD8186 monotherapy suggesting that inhibition of the PI3K/AKT pathway may be sufficient to sensitise cells to the AZD5991 combination. The cells lines showing greater combination benefit as determined by HSA score tended to be enriched for relevant pathway mutations, but again activity was not exclusively seen in pathway mutated cells. When comparing capivasertib/AZD8186 sensitive and resistant cell lines, the HSA score of the sensitive cell lines were significantly higher than the resistant models (Fig. 7C, D).

Collectively the data suggests that the combination is broadly active and not dependent on strong monotherapy activity of either compound.

### Mcl-1-AKT/PI3Kβ inhibitor combination induces apoptosis through a BAK-dependent mTORC1 independent mechanism

**Fig. 8:**
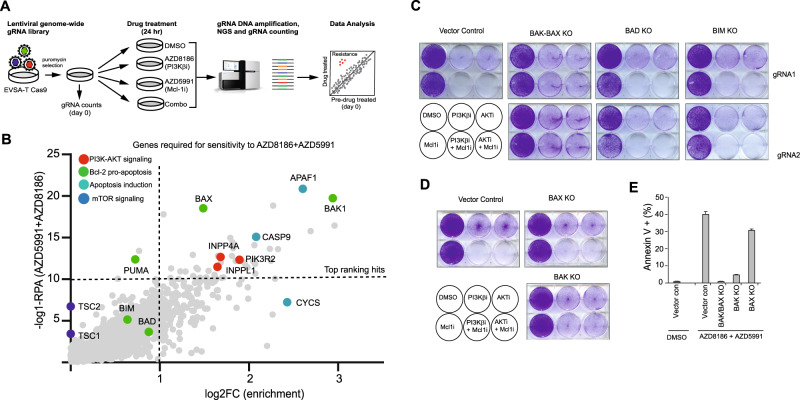
BAK1 is a key mediator of Mcl-1 and PI3K/AKT inhibitor combination response. **A** Outline of genome-scale CRISPR screening approach used to identify gene KOs that confer resistance to combination of AZD8186 + AZD5991 in EVSA-T cells. **B** Volcano plot showing gene-level values of log_2_ gRNAs FC and -log_10_ RRA obtained from the screen. Top ranking hits were determined as ≥10 -log_10_RRA and ≥1 log_2_FC in AZD8186 + AZD5991 treated cells versus control. Representative genes are highlighted. **C** Crystal violet staining of EVSA-T *BAD*, *BIM*, *BAK-BAX* KO and vector control cells treated with the indicated drugs. **D** Crystal violet staining in EVSA-T *BAK* and *BAX* KO and vector control cells treated with the indicated drugs. **E** Annexin V-staining in EVSA-T *BAX*, *BAK* single or double KO and vector control cells, treated with the indicated drugs.

The combination of Mcl-1 inhibition with AZD8186 or capivasertib rapidly induced apoptosis (<1 h) in PTEN-deficient BC cells (Fig. 4). This suggests that efficacy is likely triggered by direct signalling or post-translational modification, rather than through changes in protein expression. Inhibition of signalling pathways is often associated with modulation of apoptotic pathway genes. For example the PI3K/AKT pathway controls phosphorylation of Bad and downregulates Mcl-1 expression [40, 41]. However, western blot analysis of cells treated with capivasertib or AZD8186 alone or in combination with AZD5991 did not detect significant changes in expression (or activity) of the Bcl-2 family members (both pro and anti-apoptotic) at a timepoint where cleaved PARP is evident (Supplementary Figs. 7, 8), suggesting a novel Mcl-1 priming mechanism by PI3K/AKT inhibition. Therefore, we performed a genome-wide CRISPR KO screen to identify genes that when deleted prevent sensitivity to the combination of Mcl-1 with AKT/PI3K inhibition. Cas9-expressing EVSA-T cells transduced with the genome-wide library were treated for 24 h with either AZD8186, AZD5991 or the combination (Fig. 8A). A number of genes were significantly enriched in the combination-treated cells alone, indicating these genes are required for sensitivity to the combination (Fig. 8B and Supplementary Fig. 9). Top ranking genes included genes critical for intrinsic apoptosis: *APAF1*, *BAX*, *BAK* and *Caspase 9*. *BAX* and/or *BAK* are required to initiate cytochrome c release and apoptotic cell death. Importantly, the screening data showed that loss of *BAK* conferred stronger resistance than loss of *BAX*, while loss of none of the classic Bcl-2 pro-apoptotic genes (e.g. *BIM, PUMA, BAD or PMAIP1*(*NOXA*)) reduced sensitivity to the combination, although loss of *BIK* had a weak effect (Fig. 8B, Supplementary Fig. 9D). Consistent with previous observations (Fig. 5), *INPPL1* and *PIK3R2* loss promoted resistance to the AZD5591 + AZD8186 (Fig. 8B), whereas *TSC2* loss did not. This data further supports that inhibition of AKT signaling, but not mTORC1 signaling, is required for sensitivity to the combination of Mcl-1 inhibition with capivasertib or AZD8186.

To confirm the critical role of *BAK* in mediating the effect of the AZD8186 and AZD5991 combination, EVSA-T cells with *BAX-BAK* double KO (dKO), single *BAK*, *BAX*, *BAD* and *BIM* KOs were generated and sensitivity to the combinations of capivasertib or AZD8186 with AZD5991 tested in proliferation assays. *BAK* KO and *BAK-BAX* dKO strongly prevented a decrease in cell viability whereas *BAX, BAD* and *BIM* KO did not (Fig. 8C, D), suggesting that cell death induced by the combination is independent of *BAD*, *BIM* and *BAX*. In line with this, loss of *BAK* also prevented the induction of apoptosis following treatment with the AZD8186 and AZD5991 combination; however, some additional prevention of apoptosis was observed with the *BAX-BAK* dKO, suggesting a minor contribution from *BAX*. (Fig. 8E). These data indicate that *BAK*, but not *BAX*, is a critical mediator of sensitivity to the combination of Mcl-1 inhibition with PI3K/AKT inhibition.

Collectively, our findings show that PI3K/AKT inhibitors prime breast cancer cells for Mcl-1 induced cell death and this is dependent on the ability to suppress AKT signalling and is independent of downstream mTORC1 signaling. These results indicate that the combination of PI3Kβ/AKT inhibitors with a Mcl-1 inhibitor could be an effective therapeutic strategy to enhance treatment response and overcome mTORC1-driven acquired resistance to PI3K pathway inhibitors in *PTEN*-deficient breast cancer.

## Discussion

We have performed multiple CRISPR screens to identify and compare factors that limit or enhance efficacy of PI3K versus AKT inhibitors in PTEN null breast cancer cells. Multiple points of regulation of the PI3K-AKT pathway were identified. We find that resistance to both agents is mediated by activation of mTORC1 signalling, albeit through different factors. Genes that specifically influence sensitivity to PI3Kβi, reveal new mechanisms cells may utilise to reactivate the pathway. Despite the complexity, activity of both PI3Kβi and AKTi can be enhanced by targeting the apoptotic machinery with MCL-1, even in cells rendered resistant to PI3Kβi and AKTi monotherapy treatment.

Sensitivity to both PI3Kβ and AKT inhibitors in PTEN null tumor cell lines is limited by re-activation of PI3K/AKT/mTORC1 signaling, with both common and unique drivers of resistance to both inhibitors. However specific PI3K-pathway modulators can also drive resistance to PI3Ki versus AKTi. *TSC1*/*2* and *STK11* loss which regulate mTORC1 and AMPK respectively induced resistance to both PI3Kβi and AKTi. In contrast loss of *INPPL1* or *PIK3R2* limited PI3Kβi efficacy, possibly by restoring PI3K/AKT signalling through increased PIP3 lipids or activation of RTKs. Amino-acid dependent or mTORC1 signalling was associated with AKTi resistance as GATOR1 complex sub units *DEPDC5*, *NPRL2*, and *DDIT4*, which regulates mTORC1 stress responses limited AKTi efficacy. This suggests mTORC1 signaling at the level of 4EBP1 or S6 maintain cell viability following AKT inhibition. Finally we also found that *LCMT1* loss can also circumvent AKT suppression by modulating Protein Phosphatase 2 A (PP2A) regulation of 4EBP1 or p70S6K [42]. Our findings confirm that mTORC1 signaling is a critical mediator of resistance to PI3Kβ and AKT inhibitors which can be activated through many mechanisms. Targeting both mTORC1 and mTORC2 mediated signalling could be a strategy to target resistance to both PI3K and AKT inhibitors, however there are no dual mTORC1/2 inhibitors in clinical development.

Loss of *TSC1/2* was a key mediator of resistance to both PI3Kβ and AKT inhibitors in *PTEN*-null BC cells. These results are in line with previous studies in *PIK3CA-*mutant breast and pancreatic cancer [27, 43]. Loss of *TSC2* causes resistance to AKT/PI3K inhibitors through activation of mTORC1 independent of PI3K/AKT signaling. The role of PIK3R2 and INPPL1 in PI3Kβi resistance is intriguing as neither gene has been linked with PI3K/AKT pathway resistance. PIK3R2 encodes the P85β regulatory subunit of PI3K, (distinct from the tumor suppressor *PIK3R1* which encodes the P85α subunit). Over-expression promotes tumour growth [44] and invasion through SGK1 and Axl signalling, with P85β/p110α increasing PI(3,4,5)P3 generation from PI(4,5)P2 [45]. In the *PTEN*-deficient BC cell lines, *PIK3R2* mediated PI3Kβ resistance was not reversed by PI3Kα/δ (AZD8835) suggesting resistance is PI3K subunit independent. In contrast PI3Kβi resistance via *INPPL1* (SHIP2) which controls phospholipid levels by hydrolysing PI(3,4,5)P3 to PI(3,4)P2 was partially reversed by PI3Kβi and PI3Kα/δi, but was independent of PI3Kβi modulation of AKT activation. We have not determined the precise mechanisms by which *INPPL1* and *PIK3R2* loss mediates resistance however the mechanisms of resistance appear more complicated than merely regulation of pathway reactivation through PI3K signalling.

PI3K/AKT pathway inhibitors are used in combination due to limited single agent activity. Pathway feedback reactivation is a dominant feature associated with resistance [23, 24]. In *PTEN* null tumors, PI3Kβ inhibition drives feedback through PI3Kα which is reversed by combining PI3Kβi and PI3Kαi [24]. In PI3Kα mutant tumour cells, reactivation following PI3Kα inhibition can occur through loss of *PTEN* [28, 29] and activating PI3Kβ [23]. Combined PI3Kα and PI3Kβ inhibition prevents feedback reactivation. In *PTEN-*null cell lines combined mTORC1/mTORC2 and PI3Kβ inhibition also gives sustained tumour growth suppression [26]. For AZD8186, deletion of *ERBB2*, *ERBB3* and *PIK3CA* increased sensitivity consistent with the resistance mediated by PI3K/AKT pathway reactivation [26]. In contrast, for capivasertib deletion of genes required for mTORC1 signaling were the strongest sensitizers (such as LAMTOR subunits). Collectively we provide mechanistic proof of the importance of pathway reactivation as a primary driver of resistance to PI3K/AKT pathway inhibitors, but importantly elucidate how this can be achieved through changes at different nodes in the pathway.

Non PI3K pathway related genes were identified that can be targeted to enhance sensitivity to AKTi and PI3Ki in *PTEN*-null cells including Mcl-1 and Bcl-XL. Treatment with AZD5991 (Mcl-1 inhibitor) and AZD4320 (Bcl2/XL inhibitor) both enhanced efficacy, though Mcl-1 inhibition was more effective inducing rapid apoptosis implying direct modulation. Mcl-1 inhibition combined with both capivasertib and AZD8186, but not rapamycin, implying a specific AKT mediated effect. The Mcl-1 combined with capivasertib had broader activity than AZD8186, with activity in *PTEN*-null mutant lines and then other PI3K/AKT pathway mutations, such as *PIK3CA* mutant, suggesting sensitivity to the combination is generally associated with increased PI3K pathway activation. The activity in *TSC2* KO cells suggests that cells with mTOR-mediated resistance to PI3Ki and AKTi may be susceptible to the combination, for example when tumors progress following PI3Kα or AKT inhibitor treatment. Further work is required to predictive biomarkers or features that may discriminate sensitive and insensitive breast tumours.

The combination with Mcl-1i appears to be primed directly by inhibition of the PI3K-AKT pathway. The combination was not active in situations where AKT could not be inhibited, for example when combined with PI3Kβi in cells lacking INPPL1 and PIK3R2. The combination was however effective in these cells when combined with AKTi. CRISPR KO and biomarker studies clearly identified BAK but not BAX mediated apoptosis dependent on Caspase 9 driven cell death. We observed no regulation of Mcl-1 and Bim protein expression, or other apoptotic regulators (such as BAD) over the time course through which the combination induced apoptosis consistent with a more direct priming of Mcl-1 induced through PI3K/AKT inhibition. The concept of synergy between PI3K pathway inhibitors and the Bcl-2 family members has previously been reported in several settings, including DLBCL and AML with different mechanisms observed to drive the effect [46, 47]. In AML cells combining the AKT inhibitor ipatasertib (GDC-0068) with venetoclax (Bcl-2 inhibitor) [47], resulted in cell death through *BAX*, possibly through direct phosphorylation of BAX by AKT increasing association with mitochondria [48]. In our current study deleting *BAX* had only a minor effect on sensitivity to the combination. Ipatasertib can induce apoptosis through PUMA regulation [49] but again there was no effect on PUMA in *PTEN*-deficient tumor cells. Therefore, in *PTEN*-deficient BC cells, synergy between AZD8186/capivasertib and AZD5991 depends on *BAK* and is differentiated from the effects observed other studies. BAK and BAX can form BAK specific, BAX specific and mixed oligomers on mitochondria that all mediate mitochondrial lysis. BAK oligomers are the fastest to form [50] which would be consistent with the speed of apoptosis induction with the combination. How AKT inhibition influences BAK formation was not fully defined but the data suggests a direct mechanism. Further studies would be required to explore this in more detail.

In summary, our findings show resistance to the PI3Kβ inhibitor AZD8186 and AKT inhibitor capivasertib is mediated by specific genetic drivers, but with a common output resistance mechanism; namely the activation of mTOR signaling. Moreover, mTOR-mediated PI3Kβ and AKT inhibitor resistance can be reversed by combined treatment with an Mcl-1 inhibitor, suggesting this may be an effective combination treatment for patients progressing on PI3K or AKT inhibitors.

## Methods

### Cell culture and compounds

BC cell lines (EVSA-T (DSMZ), HCC70 (ATCC) and ZR-75-1 (ATCC)) were cultured in RPMI (ThermoFisher) supplemented with 10% FBS (ThermoFisher) and 1% GlutaMax (ThermoFisher). HEK293T cells were cultured in DMEM supplemented with, 10% FBS and 1% Glutamax (ThermoFisher). All cell lines used in this study were authenticated using STR fingerprinting and negative for mycoplasma. Cells were cultured at 37 °C under 5% CO_2_. All compounds used in this study (AZD8186, capivasertib, AZD5991, Rapamycin, AZD2014, AZD8835, AZD8931, AZD4320, AZD9496) were synthesized at AstraZeneca. For in vitro experiments, all inhibitors were dissolved in DMSO to a concentration of 10 mM and stored at −80 ^o^C. Unless stated otherwise, EVSA-T cells were treated 250 nM AZD8186, 1 µM capivasertib and 50 nM AZD5991; HCC70, 100 nM AZD8186, 500 nM capivasertib and 200 nM AZD5991; ZR-75-1, 100 nM AZD8186 and 500 nM capivasertib.

### Plasmid and lentivirus production

All plasmids used in this study are listed in Table S4. Plasmids expressing individual gRNAs were created as described previously. gRNA-expressing and Cas9-expressing lentivirus were produced as previously described [34, 51].

### Generation and validation of Cas9-expressing cell lines

Cells were transduced with a lentivirus produced from pKLV2-EF1a-Cas9Bsd-W vector [34]. 72 h after transduction, cells were selected with blasticidin (ThermoFisher) and then single sorted into 96-well plates using serial dilution. Clonally derived lines were further expanded and analysed for Cas9 cutting activity using a Cas9 reporter assay as previously described in [34]. Briefly, cells were transduced separately with lentivirus produced with pKLV2-U6gRNA5(Empty)-PGKBFPGFP-W and pKLV2-U6gRNA5(GFP gRNA)-PGKBFPGFP-W. Seventy-two-hour after transduction, the ratio of BFP and GFP-BFP double-positive cells analysed using flow cytometry using LSRFortessa instrument (BD) and resulting data analysed using FlowJo. Cas9 activity in cells (%) was calculated as (BFP-single positive cells) / (total number of BFP^+^ cells). All Cas9-cell lines used in this study had genome-editing Cas9-activity >90%.

### CRISPR-KO screen – AZD8186/capivasertib resistance and sensitivity

Screens were performed using Yusa Human CRISPR library V1 (Addgene #67989) [34] which targets 18,009 genes with 90,709 sgRNAs. Thirty million Cas9-expressing cells (EVSA-T, HCC70 and ZR-75-1) were transduced in triplicate with the lentiviral library at a multiplicity of infection of 0.3, which resulted in a library coverage of 100x. Three days after transduction, puromycin (Sigma–Aldrich) was added to the media for 4 days to kill non-transduced cells. Selected cells were cultured for a further 7 days and maintained at a minimum library coverage of 750x. For each replicate, a baseline (pre-drug treatment) sample of 68 million cells was collected. Cells were treated with vehicle control (DMSO), AZD8186 and capivasertib for 12–21 days with fresh media and compound replaced every four days. For each cell line, the exact length of drug treatment corresponded to 5–6 cell doublings of DMSO-treated cells. Concentrations of compounds used in screens were 250 nM AZD8186 and 1 µM capivasertib for EVSA-T; 100 nM AZD8186 and 500 nM capivasertib for HCC70; 50 nM AZD8186 and 500 nM capivasertib for ZR-75-1. DMSO-treated cells were maintained at 750x library coverage throughout the screen. At the end of drug selection, 68 million cells from each of the different treatment arms were pelleted and used for genomic DNA extraction. In total, 12 samples were collected per cell line (baseline, DMSO, AZD8186, capivasertib x 3 replicates).

### CRISPR-KO screen – AZD8186 + AZD5991 resistance

A mutated EVSA-T CRISPR library was generated as same as above in triplicate. Ten days after library transduction, cells were plated at 50 × 10^6^ cells per flask (Falcon 353144). On the following day, cells treated with DMSO (1 flask), 50 nM AZD5991 (2 flasks), 250 nM AZD8186 (1 flask) and a combination of 50 nM AZD5991 and 250 nM AZD8186 (10 flasks) for 20 h and then surviving cells were collected for genomic DNA extraction.

### Illumina sequencing of gRNAs and analysis of data

Genomic DNA was extracted and gRNA sequenced as described previously [34]. Single-end Illumina sequencing reads of 19 nucleotides were counted using in-house software. Significantly enriched or depleted genes in drug-treated samples were determined by comparing gRNA read counts in baseline (pre-drug treatment), DMSO-treated and drug treated samples from three independent technical replicates using MAGeCK. Functional protein interaction network analysis was performed using data from the STRING database. Adobe Illustrator was used to manually create interaction networks from STRING data (in Figs. 1, 3).

### gRNA competitive proliferation assay

For competitive proliferation assays in EVSA-T cells, BFP-labelled CRISPR KO cells were mixed with GFP-labelled control vector cells at 1:3 ratio (for resistance phenotypes) or 1:1 ratio (for sensitisation phenotypes) and treated with 250 nM AZD8186, 1 µM capivasertib or DMSO. Compound was replenished and DMSO-treated cells split every 4 days. The relative percentage of BFP+ or GFP+ cells was determined using LSRFortessa instrument (BD) at day 0 and after 14 days of drug treatment. Results are relative to day 0. FACS data was analysed using FlowJo software.

### Crystal violet sensitivity and resistance assays

For sensitivity assays, cells were seeded at 50,000 (EVSA-T) and 50,000 (HCC70) cells per well of 6-well plates and immediately treated with appropriate concentrations of compounds for 4 days. For resistance assays, cells were seeded at 10,000 (EVSA-T) cells per well of 12-well plates and immediately treated with appropriate concentrations of compounds for 9 days. Media and compounds were replaced at day 4. At the end of drug treatment, cells were fixed and stained with crystal violet. Plates were then imaged using a digital scanner. All experiments were performed at least two times. Representative experiments are shown. All images shown in each panel were obtained from the same experiment. To quantify crystal violet staining, stained plates were scanned and images obtained using a Gelcount Imager (Oxford Optronix). The images were then imported into ImageJ (Fiji) and analysed to determine the % of each well covered in stain using Binary and Area fraction functions. Average data from 3 replicate experiments were plotted in PRISM.

### Apoptosis assays

EVSA-T cells were seeded into 6-well plates and, on the following day, treated with appropriate concentration of compounds. Apoptosis was then measured by Annexin V-APC (BioLegend) or western blotting for full length and cleaved-PARP.

### Western blot analysis

Western blots were performed as described previously [52]. Briefly, cells were lysed in RIPA buffer supplemented with phosphatase (Sigma–Aldrich) and protease inhibitors (Sigma–Aldrich) and equal amounts of protein were loaded and separated by SDS-PAGE and transferred onto a PVDF membrane. Membranes were probed with primary antibody then with appropriate horseradish peroxidase-conjugated secondary and incubated with ECL. Antibodies obtained from Cell Signalling: AKT-T (#9272), pAKT-Ser473 (#4060), pAKT-Thr308 (2965), S6 (#2217), pS6-Ser235/236 (#2111), PRAS40 (#2691), pPRAS40-Thr246 (#13175), TSC2 (#4308), PARP (#9532, #9542), MCL1 (#9429), BAD-T (#9239), BAD-P Ser 136 (#4366), Bcl-XL (#2764), Bcl-w (#2724), BAX (#5023, #2772), BAK (#12105, #6947), Bim (#2933), BID (#2002), Puma (#4976), SHIP2 (#2839). Other antibodies used were Bcl-2 (ab32124, Abcam), and HRK (#ab45419, Abcam) Actin (A2228, Sigma), Vinculin (#V9132, Sigma) PIK3R2 (#A302-593A, Bethyl Laboratories), Caspase-3 (#14220). Secondary antibodies used were HRP-linked anti-rabbit IgG (CST#7074,1:2000) and HRP-linked anti-mouse IgG (CST#7076, 1:2000).

### High-throughput combination screen

Cells were dispensed into 1536 microwell plates for 24 h prior to dosing with the test compounds. Following 72 h of treatment cell viability was measured using Cell-Titer Glo 2.0 (Promega) and quantification performed using a luminescence microplate reader. The seeding density of each cell line was optimised to ensure they remained in the growth phase throughout the duration of the assay. For combination screening each compound was tested across 7 dose points with a half-log dilution series in a matrix format (49 wells). Monotherapy responses were modelled as a logistic curve with 2 parameters: shape and position. Experimental responses were limited to between 0 and 100% of cell viability. Using a multilevel model fitted with the R package nlme, the shape parameter varies across cell lines while the position is allowed to vary for each drug resulting in a unique curve for each cell line/drug pair. This model allows curves to be fitted even for unresponsive cell lines and the resulting parameters (IC50, AUC) to be used in biomarker analysis [53]. Drug combination responses were assessed using the Highest Single Agent model [54], whereby a combination of Drug 1 and Drug 2 is defined as synergy if the effect of the combination (*E*_1,2_) is larger than the effect of either Drug 1 (*E*_1_) or Drug 2 (*E*_2_) alone, whichever is the larger. Cell lines used were as indicated in the cell models passport database (https://cellmodelpassports.sanger.ac.uk/) and are routinely verified by STR and screen for mycoplasma.

$$Synergy:E_{1,2}\, > \,max\left( {E_1,E_2} \right)$$

HSA excess is calculated by subtracting the highest effect of either single agent from the combination response (*E*_1_). Here, an HSA excess > = 0.1 indicates the combination shows synergy.

$$HSA\;excess:E_{1,2} - max\left( {E_1,E_2} \right)$$

### Small combination screen

Small molecule inhibitors specific for the target genes of interest were selected and screened for synergistic effects with AZD8186 and capivasertib in an in vitro assay of proliferation following the protocol previously published [55]. EVSA-T, HCC70 and ZR-75-1 cells were seeded between 1000–8000 cells per well in 384-well plate and 24 h after plateing cells were treated for 120 h with a 6 point log dose response (0.03–3 µM) of compounds as monotherapy and in combination matrix (6 × 6) using an Echo 555 acoustic dispenser (Labcyte). An imaging-based assay utilising the cell impermeable nuclei acid dye Sytox Green (ThermoFisher) and saponin (Sigma–Aldrich) to permeabilise the cells was used to quantify live cell number. To measure the effect of compound treatment on cell proliferation and cell death over the treatment period the Sytox Green assay was used at Day 0 (pre-treatment) and at 72 or 120 h (post-treatment). Percentage growth was calculated from the live cell number. Experiments were performed in triplicate. Two- dimensional dose response matrix and curve fitting were processed in the combination extension of Genedata Screener 13™ (Genedata AG) following the methodology previously described [55]. Combination activity (synergism) was calculated using the Loewe dose-additivity model. A synergy score cut-off >5 was used to identify combinations of interest. Compounds used in drug combination screen: AZD2014, AZD8835, AZD8931, AZD5991, AZD4320, AZD9496 [56–61].

### In vivo xenograft

Female athymic nude-*Foxn1*^*nu*^ mice (Envigo) were group housed under specific pathogen-free conditions in individually ventilated Cages (Techniplast) at Alderley Park (England, United Kingdom). Mice had access to water and food *ad libitum*. Experiments were conducted in 8- to 12-week-old female mice in full accordance with the United Kingdom Home Office Animal (Scientific Procedures) Act 1986. Group sizes were determined using a statistical powering tool. Animals were transplanted subcutaneously with HCC70 cells. 1 × 10^6^ cells in 50% Matrigel were injected on the left flank of the animals. When tumours reached a volume of ~200–300 mm^3^, animals were randomised by tumour volume into groups of 9 and treatment commenced. Animals were dosed with vehicle, AZD5991 (60 mg/kg QW), AZD8186 (66.6 mg/kg QD), capivasertib (130 mg/kg BID; 4 days on, 3 days off) or in combination. QW = once weekly; QD = once daily; BID = twice a day. Capivasertib and AZD8186 were given peroral and AZD5991 by intravenous bolus at a rate of 10 ml/kg. All animal work was conducted according to AstraZeneca’s Global Bioethics Policy, in accordance with the PREPARE and ARRIVE guidelines.

Tumors were measured twice weekly by caliper and the volume of tumours calculated using elliptical formula (pi/6000 × width × width × length). AZD8186 was formulated as milled nano-suspension in Polyvinyl pyrrolidone (Kollidon K30 ex BASF) 1.34% w/v with Aerosol OT (Dioctyl sodium sulfosuccinate – ex Cytec Ind.) 0.067% w/v at 100 mg/ml and then diluted to 6.6 mg/ml in WFI (Water for injection) for dosing. Capivasertib was formulated in 10% DMSO, 25% Kleptose solution. AZD5991 was formulated in 30% HP-β Cyclodextrin adjusted to pH 9-9.5 with 1 N Meglumine. The relative tumour volume change from the start of the treatment was assessed by comparison of the mean change in tumour volume for the vehicle-control and drug-treated groups.

### Pharmacodynamic studies

Total tumour lysates were generated in the Cell Extraction Buffer (Invitrogen) supplemented with DDT (1 mM, Sigma–Aldrich), protease inhibitors 2 and 3 (1:100, Sigma) and phosphatase inhibitor (1:200, Sigma) using the MP Biomedicals FastPrep-24 machine (three 1 min cycles of 6.5 m/s, with 5 min on ice intervals). Samples were sonicated for 30 s (Diagenode, high frequency setting) and incubated in ice for another 15 min. Samples were then centrifuged for 10 min at 13,000 rpm and supernatants were collected. Protein concentration was determined using BCA Protein Assay Kit (Pierce). For Western blot analysis, equal amount of proteins (55 µg) in NuPAGE LDS sample buffer and Reducing Agent (Invitrogen) were loaded on BisTris 4–12% gradient gels (Invitrogen) and run at 180 V in NuPAGE MES Running Buffer (Invitrogen). Proteins were then transferred onto 0.2 µm nitrocellulose membranes using iBlot system (Invitrogen). Membranes were blocked in 5% milk (Marvel) in TBST (0.05% Tween 20) for 1 h at room temperature and then probed overnight at 4 °C with primary antibodies. After the incubation, membranes were washed three times with TBST for 5 min and probed with secondary antibodies for 1 h at room temperature, followed by washing with TBST. All secondary antibodies were diluted in 5% milk (Marvel) in TBST. Signal was detected using SuperSignal West Dura Chemiluminescent Substrate reagent (Pierce) and G:Box instrument (Syngene). Bands were quantified using Genetools software (Syngene). Protein levels of each biomarker were normalised to loading control (vinculin) levels and to control vehicle group. A two-sided *t*-test was performed on logged data to determine statistical significance. *p ≤ 0.05, **p ≤ 0.01, ***p ≤ 0.001.

### PDX-93T study

Fresh tumour samples from patients with breast cancer were collected following a Vall d’Hebron University Hospital’s Institutional Research Board approved protocol and the associated written informed consent. The study was compliant with the declaration of Helsinki. Experiments were conducted following the European Union’s animal care directive (2010/63/EU) and were approved by the Ethical Committee of Animal Experimentation of the Vall d’Hebron Research Institute (Barcelona, Spain) and the Catalan Government. Experiments were ended when the tumour volume surpassed 1500 mm^3^ or a decline in mouse welfare was observed, including mouse weight loss >20%. Six-week-old female athymic nude mice (HsdCpb:NMRI-*Foxn1*^*nu*^, Harlan Laboratories) were housed in air-filtered laminar flow cabinets with a 12-hour light cycle and food and water *ad libitum*. Fragments of 50 mm tumors were implanted into mice. Animals were supplemented with 1 μmol/L 17β-estradiol (Sigma) in their drinking water. Upon xenograft growth, tumour tissue was implanted on the lower flank of new recipient mice. When tumour volume was 150 to 300 mm^3^, mice were equally distributed into the experimental groups and were treated with vehicle (*n* = 4) capivasertib (*n* = 8), AZD8186 (*n* = 2), AZD5991 (*n* = 5), or the combination of capivsertib+AZD5991 (*n* = 7) or AZD8186 + AZD5991 (*n* = 4) with the formulations and dosing schedules described in the in vivo xenograft methods. For the combination treatments, all drugs were given at the same dose and schedule as in the single treatment arms, but AZD5991 was injected on day 2 one hour after the administration of AZD8186 or the first dose of the day of capivasertib.

### Statistical analyses

Data shown in graphs are mean + /− SD, unless stated otherwise. Most statistical analyses (*t*-test, multiple comparisons) were performed using GraphPad Prism. Significance values are as follows ns *p* > 0.05, *p ≤ 0.05, **p ≤ 0.01, ***p ≤ 0.001,****p < 0.0001. For screening data, significantly enriched or depleted genes in drug-treated samples were determined by comparing gRNA read counts in baseline (pre-drug treatment), DMSO- or drug-treated samples from three independent technical replicates using MAGeCK statistical package. Boxplots display the median, first and third quartiles (lower and upper hinges), the largest value smaller and the smallest value larger than 1.5× interquartile range (upper and lower whiskers).

## Supplementary information

Supplementary Figure legends and Tables

Supplementary Figures
