# Supplementary material for: AKT-mTORC1 reactivation is the dominant resistance driver for PI3Kβ/AKT inhibitors in PTEN-null breast cancer and can be overcome by combining with Mcl-1 inhibitors
Source: Oncogene. 2022 Oct 14;41(46):5046–60. doi: 10.1038/s41388-022-02482-9 (PMC9652152; doi:10.1038/s41388-022-02482-9)
Supplement: Supplementary file 1 — Supplementary Figure legends and Tables [file 41388_2022_2482_MOESM1_ESM.docx]

**Supplementary Information Dunn at al:**

**AKT-mTORC1 reactivation is the dominant resistance driver for PI3Kβ/AKT inhibitors in PTEN-null breast cancer and can be overcome by combining with Mcl-1 inhibitors**

**Supplementary Figure Legends**

**Supplementary Figure 1. Characterisation of Cas9-expressing *PTEN*-null breast cancer cell lines used in CRISPR screens**

**(A)** High Cas9 activity was confirmed in the Cas9-expressing EVSA-T, HCC70 and ZR-75-1 cells. Flow cytometry profiles of BFP and GFP fluorescence in Cas9-expressing cells transduced with Cas9 lentiviral reporter assay (mock, no virus; BFP-GFP, control virus; BFP-GFP + gRNA GFP, gGFP-expressing virus). FACS analysis confirmed loss of GFP expression in Cas9-expressing cells transduced with BFP-GFP + gRNA GFP virus. The activity of Cas9 (%) in the cells is the fraction of cells that are BFP positive but GFP negative. **(B)** Cas9 activity does not impact AZD8186/capivasertib sensitivity in the three *PTEN*-null lines. Dose response curves of HCC70, EVSA-T, ZR-75-1 parental and Cas9-expressing cells in the presence of increasing concentrations of AZD8186 and capivasertib up to 1µM. Proliferation was measured at 72 hours using Cell-Titer Glo. The relative proliferation of vehicle control treated (DMSO) cells is set to 1.

**Supplementary Figure 2. Quality assessment of the CRISPR screening data**

**(A)** Receiver operating characteristic analysis using defined essential and non-essential gene sets, confirming high screening performance. Area under the curve was indicated in the brackets next to the cell line names. The statistical output comparing day 0 (plasmid) and the end-point culture of DMSO treated cells was used. **(B)** Gene set enrichment analysis showing screen validity in the EVSA-T screening data. Essential gene sets (RNA polymerase and ribosome) were used. **(C)** Scatter plots comparing gene depletion statistics and gene expression. Predicted dependencies, namely *AKT1* and *PIK3CB,* were confirmed. **(D)** gRNA FCs of the indicated genes, confirming *AKT1* and *PIK3CB* dependency. Each dot represents a gRNA of each replicate. *COMP, FAM184B* and *MAB21L2* were shown as a representative of non-essential genes.

**Supplementary Figure 3. Validation of TSC2, INPPL1 and PIK3R2 as AZD8186 and capivasertib drug resistance genes**

**(A-C)** Quantification of crystal violet staining of EVSA-T *INPPL1* **(A)**, *PIK3R2* **(B)** and *TSC2* **(C)** KO or Vector control cells treated with DMSO, 250nM AZD8186 or 1μM capivasertib. Two individual KO clones are shown**. (D-G)** Quantification of crystal violet staining of EVSA-T *control* **(D)***,* *PIK3R2* **(Ei,ii)**, *INPPL1* **(Fi,ii)** and *TSC2* **(Gi,ii)** KO cells treated with DMSO, 250nM AZD8186 or 1μM capivasertib in combination with DMSO, 1μM capivasertib, 100nM Rapamycin, 500nM AZD2014 and 500nM AZD8835 combinations for 10 days as indicated. All plates were quantified using ImageJ software and % confluency of each well calculated. Data are mean of 3 independent experiments ± SD. ns = P>0.05; * = P≤ 0.05; ** = P≤ 0.01; *** = P≤ 0.001; **** = P≤ 0.0001.

**Supplementary Figure 4. AZD8186/capivasertib + AZD5991 combination in *TSC2*, *INPPL1* and *PIK3R2* KO cells**

**(A-C)** Western blot analysis of PI3K pathway effectors (pAKT, pPRAS40, pS6, p4E-BP1), pNDRG1 and PARP in EVSA-T *INPPL1* **(A)**, *PIK3R2* **(B),** and *TSC2* **(C),** KO cells treated with the indicated drugs for 4 hours. Data are representative of three independent experiments. Same as Figure 6G, but data generated using two independent gRNAs (gRNA 1 and 2) are shown. **(D,E)** Lysates from control and PIK3R2, INPPL1 and TSC2 KO EVSA-T cells were analysed by western blot for PARP cleavage, Bcl-xL, Bcl-w, Mcl1, BID, Puma, Vinculin, Bax, Bax, BIM, BAD as indicated. KO cells generated with two independent guides are shown.

**Supplementary Figure 5. Body weight changes in HCC70 and PDX93T tumours following monotherapy or combination drug treatment**

**(A, B)** Mean body weight change in animals bearing HCC70 tumours over time following treatment with capivasertib, AZD8186 and AZD5991 alone and in combination as indicated. **(C)** Mean body weight change over time in animals bearing PDX96T tumours following treatment with capivasertib, AZD8186 and AZD5991 alone and in combination as indicated. Mean weight in gm is represented +/- SEM.

**Supplementary Figure 6. Analysis of pharmacodynamic biomarkers in the PTEN-deficient xenograft tumours following monotherapy or combination drug treatment**

HCC70 xenografts were treated with AZD5991 (60mg/kg QW), AZD8186 (66.6mg/kg QD), capivasertib (130mg/kg BID) and in combination. **(A-B)** The analysis of pharmacodynamics markers. HCC70 tumour xenografts were treated for two days with AZD5991, AZD8186, capivasertib and in combination and analysed for the indicated markers. Plots represent geometric mean ± SEM biomarker signal. *p<0.05, **p<0.01, ***p<0.001. n=3-5 animals. **(C)** Plot represents tumour volumes from individual animals in the PDX93T study. Capivasertib treated tumours (green) and capivasertib + AZD5991 tumours (purple) are shown.

**Supplementary Figure 7. Expression of Bcl-2 protein family following treatment of AZD5991, AZD8186, capivasertib or combinations in PTEN-deficient breast cancer cells**

Expression of Bcl-2 protein family in *PTEN*-null breast cancer cells. Western blot analysis with antibodies against the Bcl-2 family members (BAD, BAK, BAX, BID, BIM, Bcl2, Bcl-XL and MCL1), PARP (full length and cleaved) and AKT (total and pS473 and pT308) in **(A)** HCC-70 and **(B)** EVSA-T cells treated with the indicated drugs at the indicated concentration for 4 hours. Pro-apoptotic Bcl-2 family members = BAD, BAK, BAX, BID, BIM. Anti-apoptotic Bcl-2 family members = Bcl2, Bcl-XL and MCL1.

**Supplementary Figure 8. Expression of pro- and anti-apoptopic proteins following AZD8186, capivasertib and AZD5991 treatment in EVSA-T cells**

Changes in specific pro- and anti-apoptopic genes were assessed following drug treatment to determine whether priming of cell death was associated with rapid changes in proteins regulating apoptosis. EVSAT-T cells were treated with 250nM AZD8186, 1μM capivasertib, 50nM AZD5991 alone and in combination for 4 hours . Lysates were analysed by Western blotting for pAKT473, PARP cleavage, vinculin, Bcl-w, Bcl-2, Bcl-xL , Mcl-1, BID, HRK, Puma expression as indicated.

**Supplementary Figure 9. Genome-scale CRISPR screen identifies genes mediating sensitivity to AZD8186 + AZD5991 combination**

Gene-level scatter plot of -log_10_RRA vs mean log_2_FC gRNA enrichment in AZD8186 **(A)**, AZD5991 **(B)** and AZD8186+AZD5991 **(C).** Top ranking hits are defined as ≥10 -log_10_RRA and ≥1 log_2_FC gRNA in drug-treated cells versus control. Each dot represents a gene. Representative genes are highlighted. **(D)** Gene-level scatter plot of -log_10_RRA vs mean log_2_FC gRNA enrichment in AZD8186+AZD5991 of Bcl-2 family members. **(E)** List of Bcl-2 family members highlighted in **(D)**.

**Supplementary Tables**

**Table S1. Capivasertib resistance genes identified in the EVSA-T, HCC-70 and ZR-75-1 CRISPR screens**

Genes most enriched (log2FC gRNA)in capivasertib-treated vs DMSO-treated cells at the top of the ranked gene list.

| **EVSA-T (PR+ER-)**  **(** | **HCC-70 (TNBC)** | **ZR-75-1 (ER+PR-)** |
| --- | --- | --- |
| **61 genes** | **79 genes** | **48 genes** |
| PPP2R1A | TSC1 | STK11 |
| STK11 | TSC2 | TSC1 |
| TSC1 | RARG | TSC2 |
| NPRL2 | SPPL3 | RALGAPB |
| LCMT1 | NF2 | DDIT4 |
| TSC2 | ATG7 | LCMT1 |
| CMTR2 | RXRA | PPME1 |
| DEPDC5 | GORASP2 | CNOT1 |
| PELO | FOXO3 | CLASRP |
| CNOT1 | CCAR1 | ZFP36L1 |
| MTM1 | MED12 | BAP1 |
| ZFP36L2 | ZC3H4 | FIBP |
| SZT2 | ATG12 | RFXAP |
| SEC63 | HNRNPD | EIF3L |
| KEAP1 | ATG10 | USP9X |
| C12orf66 | ATG3 | HDAC1 |
| TBC1D7 | SMARCB1 | AUP1 |
| INPP4A | SEC31A | MTMR9 |
| SETD2 | TAF6L | INPPL1 |
| PPP2CA | ATG9A | C18orf8 |
| MAPKAP1 | SLC39A9 | CNOT11 |
| CMTR1 | SMG8 | SMS |
| LDB1 | NPRL2 | HDAC2 |
| MAU2 | CDK5 | STRADA |
| PPME1 | IKZF2 | RREB1 |
| ZC3H4 | TADA2B | MTMR6 |
| DDIT4 | SUPT7L | FAM13B |
| PPP2R5E | RBM47 | CSNK2A1 |
| MTCH1 | ATG4B | CDK5 |
| C18orf8 | N6AMT1 | UBE2G2 |
| MED23 | ZNF217 | PPP2R5A |
| MARK2 | SEC23IP | BAG6 |
| FAM13A | STUB1 | AMBRA1 |
| TM2D3 | MAGT1 | UBE2A |
| CSK | RASA1 | PDS5B |
| ITFG2 | WIP12 | WDR81 |
| ACVR1B | RARA | TRIM8 |
| CDKN1B | NRARP | RAB5C |
| CDK5 | STT3B | SBNO2 |
| EIF3L | SAR1A | ZBTB7A |
| MGAT1 | MGAT1 | ZC3H4 |
| MED16 | TMEM127 | NPRL2 |
| HMOX2 | KIAA1211 | WDR83 |
| GSKIP | REST | MBD6 |
| CABLES1 | DEPDC5 | DHX35 |
| ADCK3 | ARID2 | IPO5 |
| ANKRD52 | INPP5K | CABIN1 |
| PPP2R2A | TECRL | MFSD6 |
| FIBP   \| FIBELAVL1P \| \| --- \| \| ELAVL1 \| \| TRAF7 \| \| TBC1D4 \| \| ATMIN \| \| FZR1 \| \| FUBP3 \| \| PSENEN \| \| GSK3A \| \| GSE1 \| \| MTMR9 \| \| B3GNT2 \| \| INPPL1 \| | RB1CC1 |  |
| ELAVL1 | ATG5 |  |
| TRAF7 | SMG9 |  |
| TBC1D4 | RAB35 |  |
| FZR1 | NPEPPS |  |
| FUBP3 | GCN1L1 |  |
| PSENEN | OVOL2 |  |
| GSK3A | CHIC2 |  |
| GSE1 | ORMDL3 |  |
| MTMR9 | FBXW7 |  |
| B3GNT2 | SHB |  |
| INPPL1 | SEC24A |  |
|  | C12orf44 |  |
|  | SEC16A |  |
|  | RBL2 |  |
|  | HNRNPDL |  |
|  | RALGAPB |  |
|  | SMAD1 |  |
|  | BCL2L11 |  |
|  | ALCAM |  |
|  | CXCL1 |  |
|  | CXCL3 |  |
|  | ANKRD39 |  |
|  | SBNO2 |  |
|  | SPINK2 |  |
|  | RBM5 |  |
|  | ATXN7 |  |
|  | DDIT4 |  |
|  | PINX1 |  |
|  | ULK1 |  |
|  | PPEF2 |  |

**Table S2. AZD8186 resistance genes identified in EVSA-T, HCC-70 and ZR-75-1 CRISPR screena**

Genes most enriched (log2FC gRNA)in AZD8186-treated vs DMSO-treated cells at the top of the ranked gene list.

| **EVSA-T (PR+ER-)**  **(** | **HCC-70 (TNBC)** | **ZR-75-1 (ER+PR-)** |
| --- | --- | --- |
| **53 genes** | **37 genes** | **21 genes** |
| INPP4A | INPPL1 | TSC1 |
| CSK | PIGL | TSC2 |
| ELOF1 | RARG | STK11 |
| HUWE1 | PIK3R1 | INPPL1 |
| PIK3R2 | N6AMT1 | PIK3R2 |
| INPPL1 | MED12 | ZBTB7A |
| SUPT4H1 | SPPL3 | PPME1 |
| PPP2R1A | HNRNPD | PDS5B |
| LCMT1 | NF2 | GPBP1 |
| GRB2 | RXRA | HDAC2 |
| MTCH1 | ATG7 | ZNF367 |
| STK11 | DPH1 | SBNO2 |
| UBE2F | CXorf56 | HDAC1 |
| TBX3 | GORASP2 | ZFP36L1 |
| SETD2 | EPHA1 | FIBP |
| SIRT1 | TAF6L | RREB1 |
| MED23 | FBXO11 | CABIN1 |
| NPRL2 | MAPKAPK5 | GGNBP2 |
| TSC2 | PIK3R2 | LZTR1 |
| ZFP36L2 | CDK5 | TRIM8 |
| STAG2 | SMG8 | NR2F1 |
| TSC1 | SMG9 |  |
| NF2 | ATG12 |  |
| ARIH2 | SEC31A |  |
| RNF7 | TSC2 |  |
| SP1 | SEC23IP |  |
| MED16 | HNRNPUL1 |  |
| TM2D3 | PIK3R3 |  |
| PPP2CA | SLC39A9 |  |
| DEPDC5 | MGAT1 |  |
| GSE1 | CTNND1 |  |
| VCPIP1 | KIAA1211 |  |
| QRICH1 | BCL2L11 |  |
| ACVR1B | PINX1 |  |
| FIBP | SHROOM3 |  |
| FGFR4 | SHB |  |
| ADCK3 | ATG4B |  |
| NOSIP |  |  |
| NFAT5 |  |  |
| MSL3 |  |  |
| C12orf66 |  |  |
| NONO |  |  |
| ASB3 |  |  |
| CXorf56 |  |  |
| OTUD5 |  |  |
| RBM5 |  |  |
| MED25 |  |  |
| CDK5 |  |  |
| TENC1 |  |  |
| CABLES1 |  |  |
| SOCS6 |  |  |
| KIAA0195 |  |  |
| SZT2 |  |  |

**Table S3. gRNA sequences used to generate knockout cell lines in this study**

| **Gene** | **gRNA sequence** | **gRNA I from Human V1 library** |
| --- | --- | --- |
|  |  |  |
| TSC2 (1) | CCTCGACGAGTACATCGCA | TSC2_CCDS10458.1_ex4_16:2105489-2105512:+_5-1 |
| TSC2 (2) | GACGTTGATGGTGCGACAG | TSC2_CCDS10458.1_ex6_16:2106721-2106744:-_5-2 |
| TSC1 (1) | TTCATCCGGAATTAGTGAC | TSC1_CCDS55350.1_ex15_9:135796779-135796802:-_5-4 |
| TSC1 (2) | ACGTCGTTGTCCTCACAAC | TSC1_CCDS55350.1_ex17_9:135798847-135798870:-_5-5 |
| NPRL2 (1) | TCTTGTGTTCGATGCACAC | NPRL2_CCDS2826.1_ex8_3:50387210-50387233:+_5-4 |
| NPRL2 (2) | CAATTATCCAGCACAGCTA | NPRL2_CCDS2826.1_ex8_3:50387253-50387276:-_5-5 |
| INPPL1 (1) | TCACTGCGGTCGTGCGTGA | INPPL1_CCDS8213.1_ex8_11:71941297-71941320:-_5-4 |
| INPPL1 (2) | ACGGATCTGGATTACCGCC | INPPL1_CCDS8213.1_ex11_11:71942211-71942234:+_5-5 |
| NF2 (1) | CTGGCTTCTTACGCCGTCC | NF2_CCDS13861.1_ex3_22:30038246-30038269:+_10-6 |
| NF2 (2) | CTTGGTACGCAGAGCACCG | NF2_CCDS13861.1_ex5_22:30051633-30051656:+_10-8 |
| STK11 (1) | GGCTCTTACGGCAAGGTGA | STK11_CCDS45896.1_ex0_19:1207082-1207105:+_5-1 |
| STK11 (2) | AGGTGTCGTCCGCCGCGAA | STK11_CCDS45896.1_ex4_19:1220588-1220611:-_5-5 |
| PIK3R2 (1) | GCTAGAAGCATCTCGGACT | PIK3R2_CCDS12371.1_ex7_19:18273262-18273285:-_5-4 |
| PIK3R2 (2) | GGTCAGCGTGTACTCGCCC | PIK3R2_CCDS12371.1_ex7_19:18273289-18273312:-_5-5 |
| FIBP (1) | CATGTGGAAGGTGCGGTAA | FIBP_CCDS8118.1_ex8_11:65655492-65655515:+_5-4 |
| FIBP(2) | ATTCCCGAGCGCACCCGCA | FIBP_CCDS8118.1_ex8_11:65655565-65655588:+_5-5 |
| PIK3CA (1) | TTCGAACAGGTATCTACCA | PIK3CA_CCDS43171.1_ex4_3:178922296-178922319:+_5-3 |
| PIK3CA (2) | TCTCGGGATACAGACCAAT | PIK3CA_CCDS43171.1_ex7_3:178928300-178928323:-_5-5 |
| ERBB3 (1) | AGCATCGCCGGTCACACTC | ERBB3_CCDS31833.1_ex1_12:56477556-56477579:-_5-1 |
| ERBB3 (2) | CCATTGCCCAACCTCCGCG | ERBB3_CCDS31833.1_ex2_12:56478834-56478857:+_5-5 |
| ERBB2 (1) | TTACAGGCCCGAGAGCGGT | ERBB2_CCDS32642.1_ex3_17:37865687-37865710:-_5-1 |
| ERBB2 (2) | CGAATGTATACCGGCCCTC | ERBB2_CCDS32642.1_ex6_17:37866685-37866708:-_5-2 |
| BAD (1) | CTATGGCCGCGAGCTCCGG | BAD_CCDS8065.1_ex1_11:64039114-64039137:-_5-1 |
| BAD (2) | ATAGCGCTGTGCTGCCCAG | BAD_CCDS8065.1_ex1_11:64039131-64039154:+_5-2 |
| BCL2L11 (1) | AGGGGCCCCAGGTCTGAGC | BCL2L11_CCDS2089.1_ex0_2:111881399-111881422:-_17-1 |
| BCL2L11 (2) | CTCTGTCTGTAGGGAGGTA | BCL2L11_CCDS2089.1_ex0_2:111881417-111881440:-_17-2 |
| BAK (1) | CCCTACACGTCTACCAGCA | BAK1_CCDS4781.1_ex1_6:33541904-33541927:-_5-1 |
| BAK (2) | GAACTCTGAGTCATAGCGT | BAK1_CCDS4781.1_ex2_6:33543144-33543167:+_5-3 |
| BAX (1) | GACACAGACTCCCCCCGAG | BAX_CCDS12742.1_ex3_19:49459469-49459492:+_5-1 |
| BAX (2) | TTCTGACGGCAACTTCAAC | BAX_CCDS12742.1_ex3_19:49459519-49459542:+_5-3 |

**Table S4. Vectors used in this study**

| **Plasmid** | **Addgene Reference ReRRereference** | **Method used in** |
| --- | --- | --- |
|  |  |  |
| - pKLV2-U6gRNA5(Empty)-PGKBFP2AGFP-W | #67979 | Cas9 activity assay |
| - pKLV2-U6gRNA5(gGFP)-PGKBFP2AGFP-W | #67980 | Cas9 activity assay |
| - pKLV2-EF1a-Cas9Bsd-W | #68343 | Cas9 cell line generation |
| Human improved genome-wide KO CRISPR library | #67989 | CRISPR screening |
| - pKLV-U6gRNA(BbsI)-PGKpuro2ABFP | #50946 | CRISPR KO cell line generation |
| - pKLV-U6gRNA(BbsI)-PGKpuro2AGFP | #67976 | CRISPR KO cell line generation |
| - dual gRNA vector | #72666 | BAK-BAX KO cell line generation |
