# Supplementary material for: AKT-mTORC1 reactivation is the dominant resistance driver for PI3Kβ/AKT inhibitors in PTEN-null breast cancer and can be overcome by combining with Mcl-1 inhibitors
Source: Oncogene. 2022 Oct 14;41(46):5046–60. doi: 10.1038/s41388-022-02482-9 (PMC9652152; doi:10.1038/s41388-022-02482-9)
Supplement: Supplementary file 2 — Supplementary Figures [file 41388_2022_2482_MOESM2_ESM.pdf]

Supplementary Figure 1

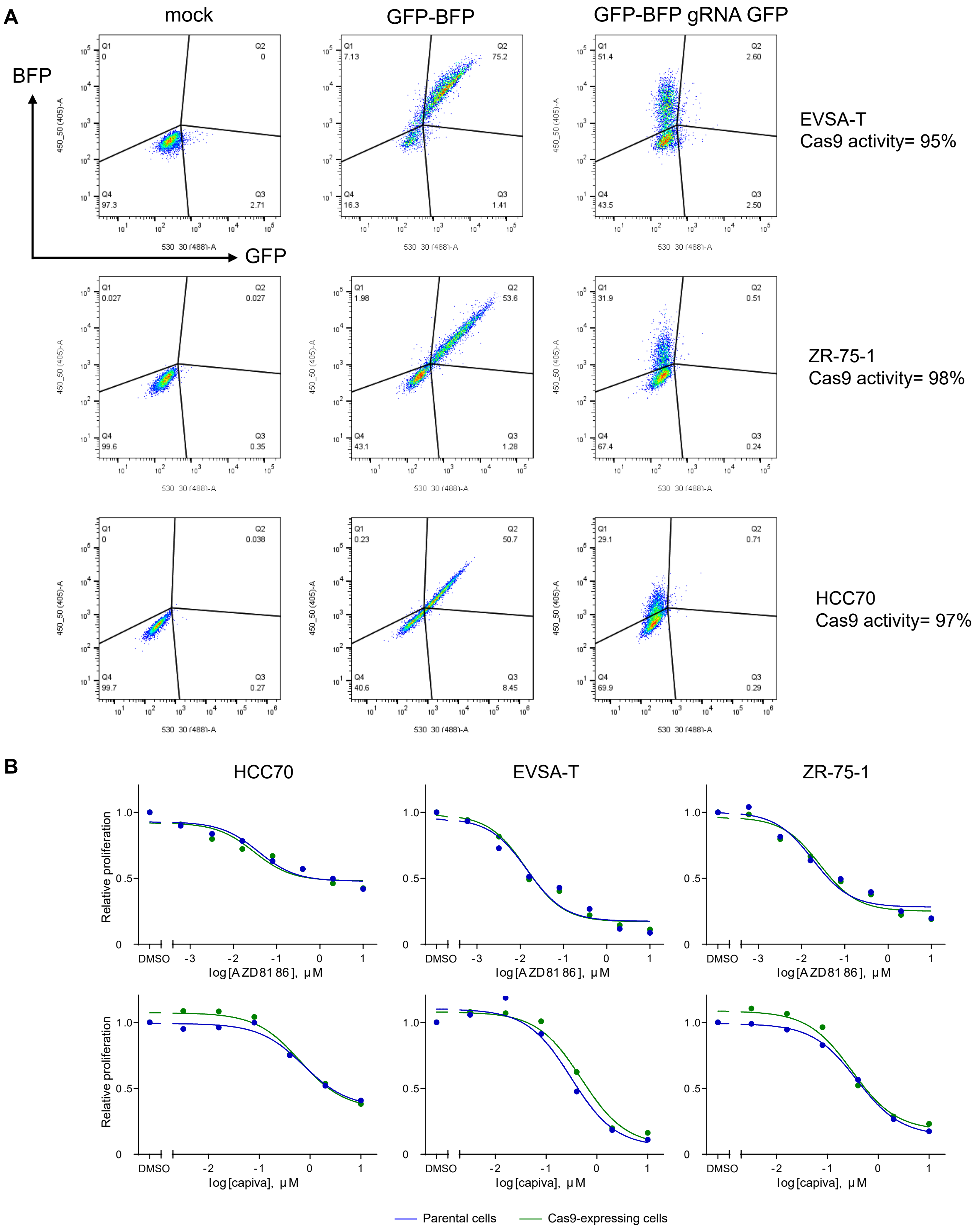

Supplementary Figure 2

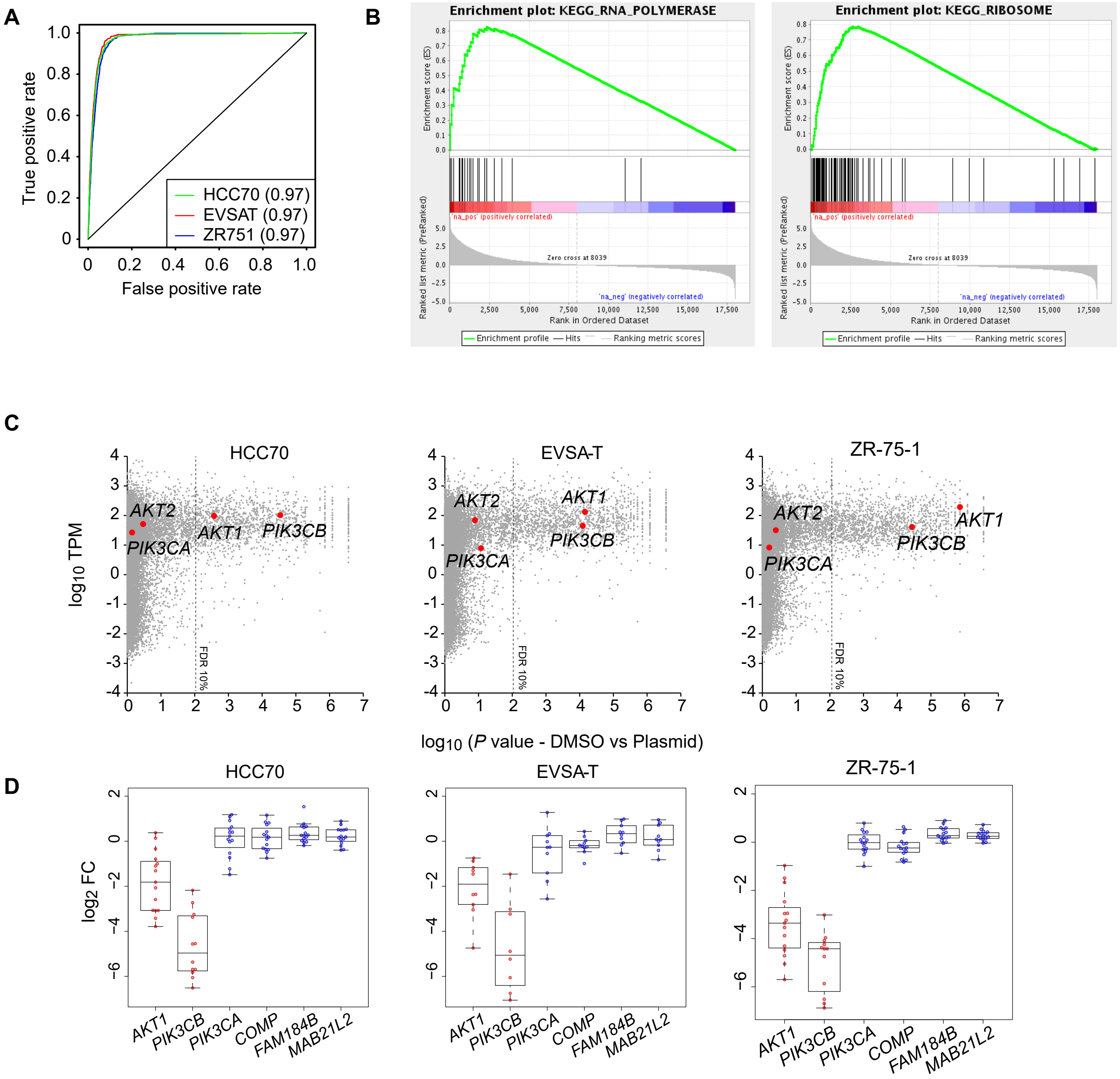

Supplementary Figure 3

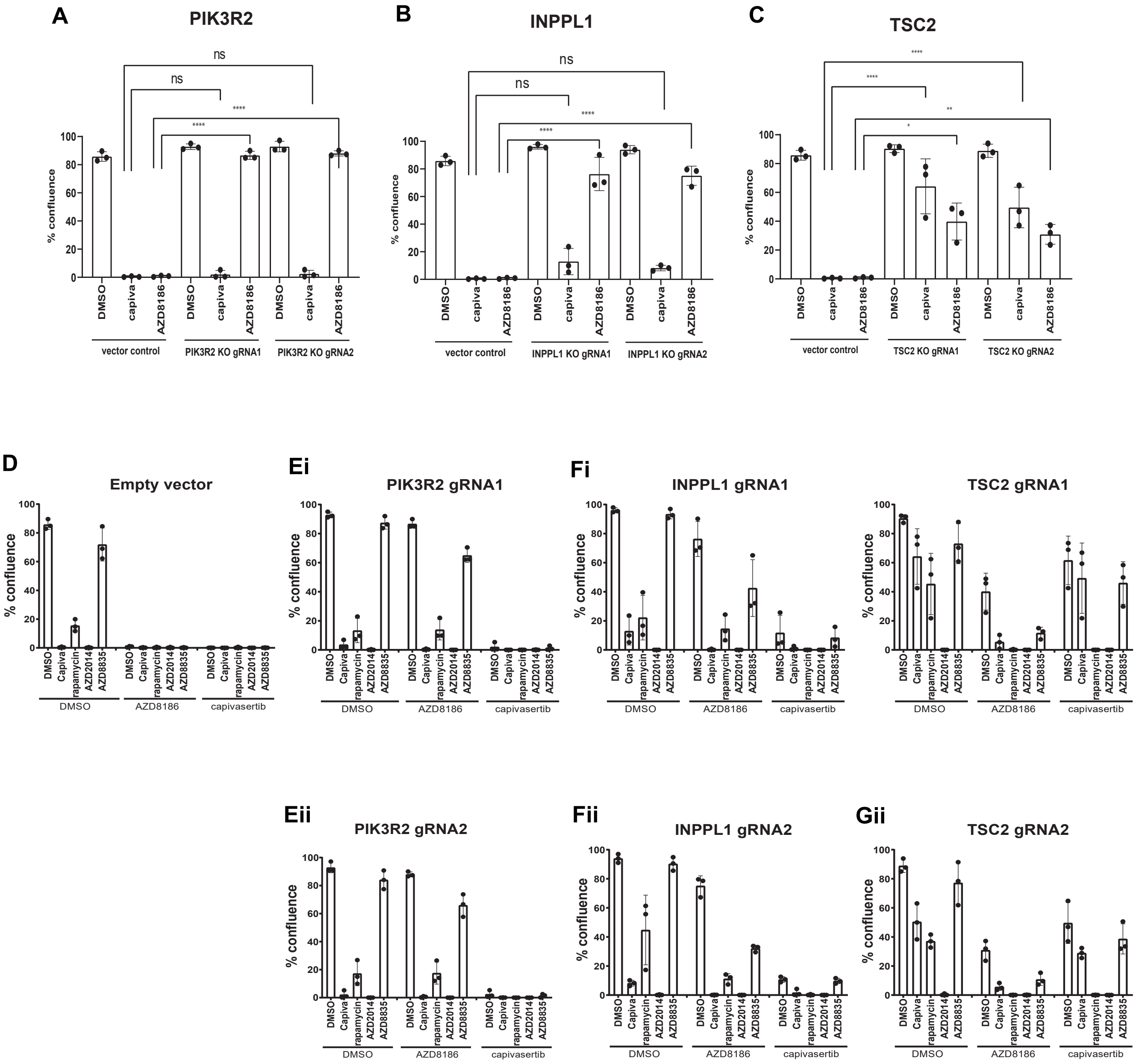

Supplementary Figure 4

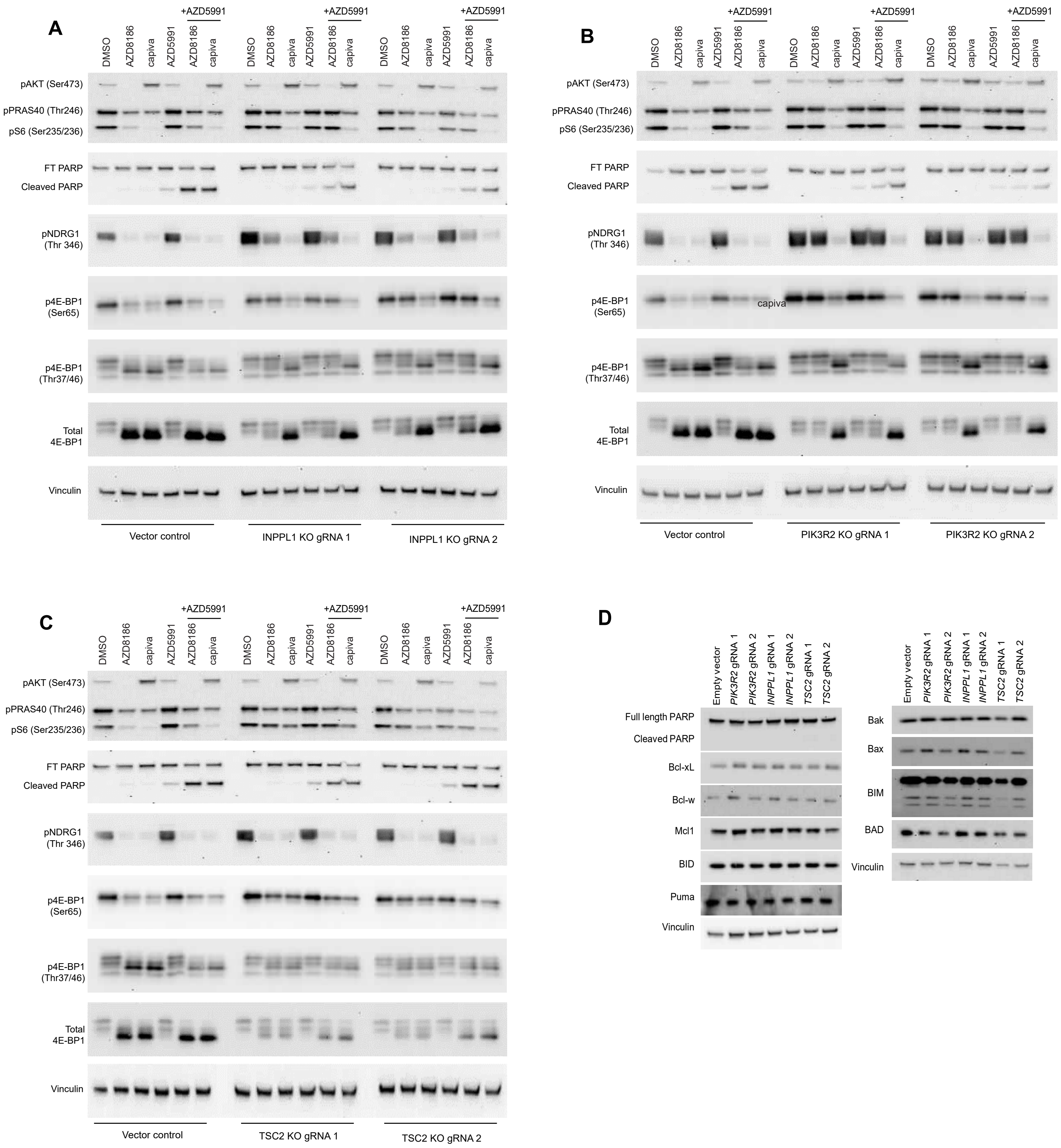

Supplementary Figure 5

A

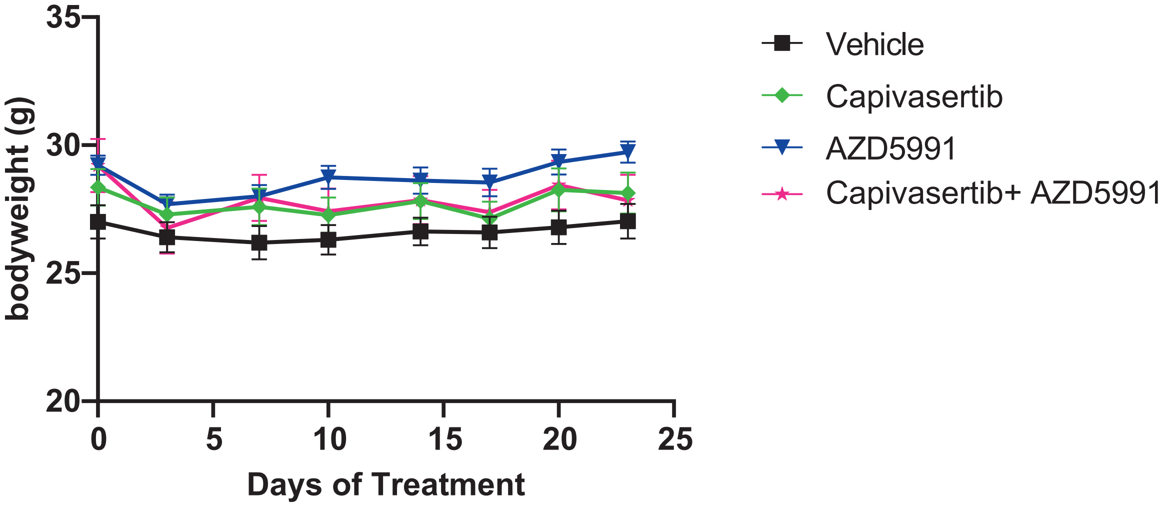

B

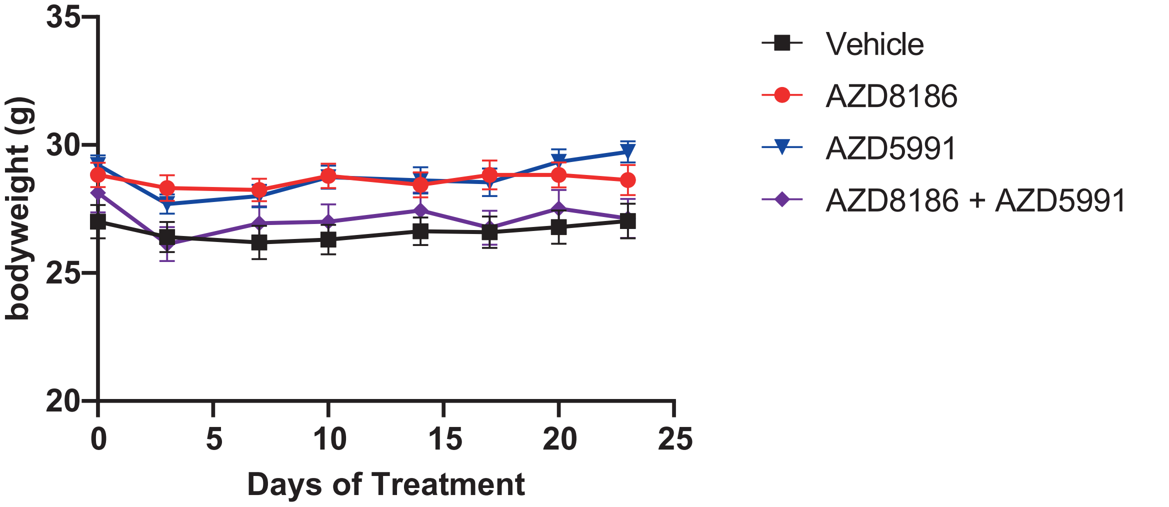

C

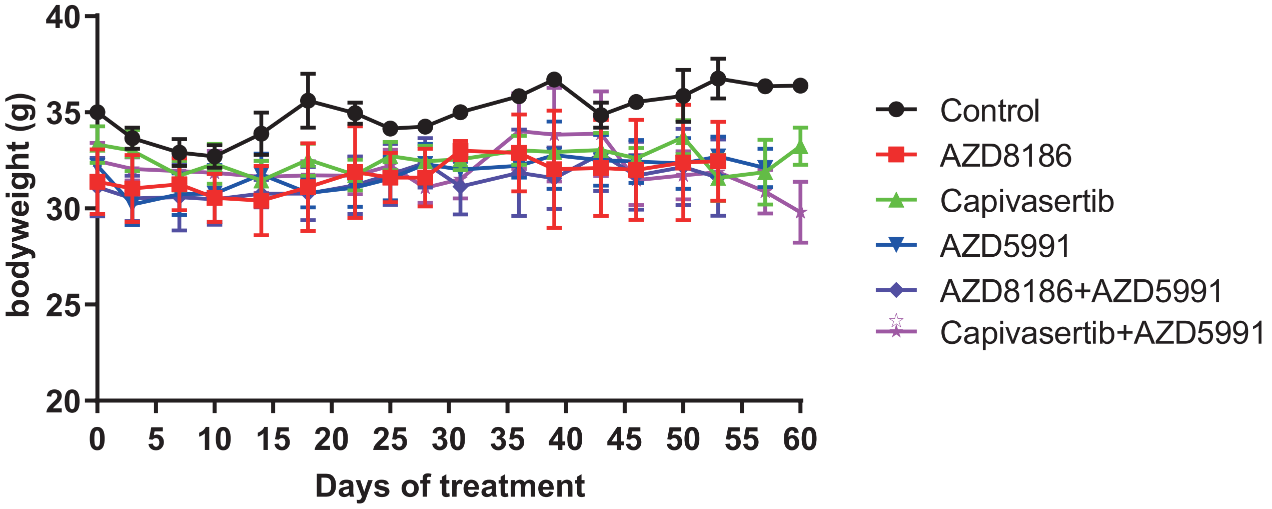

Supplementary Figure 6

A

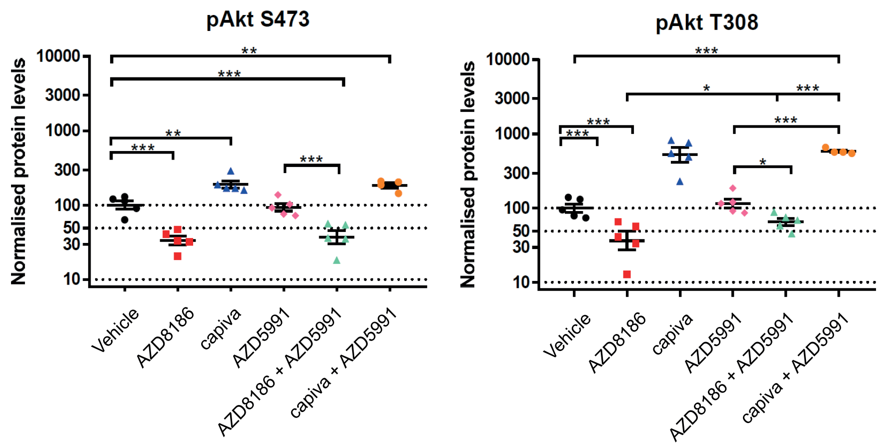

B

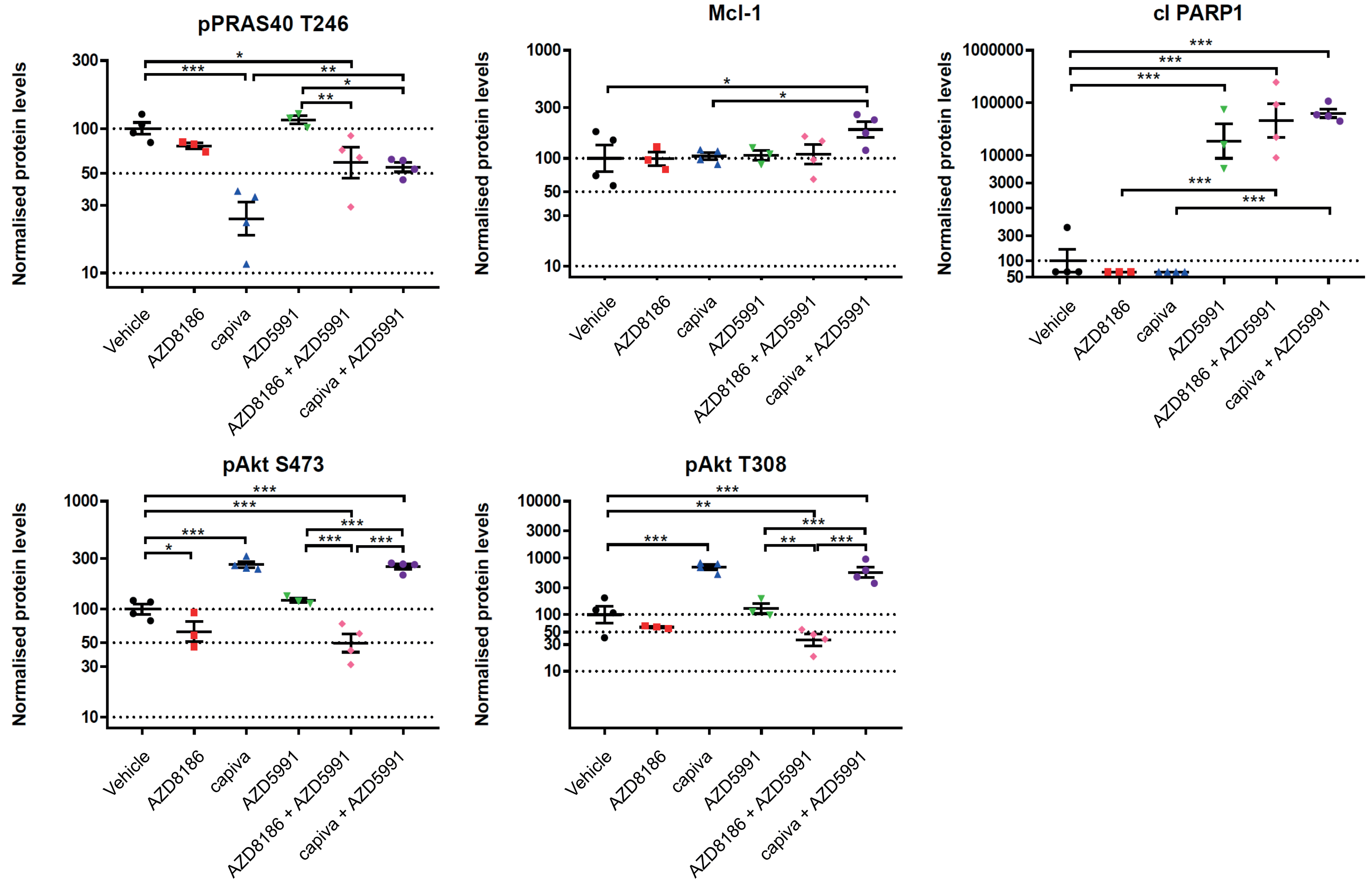

C

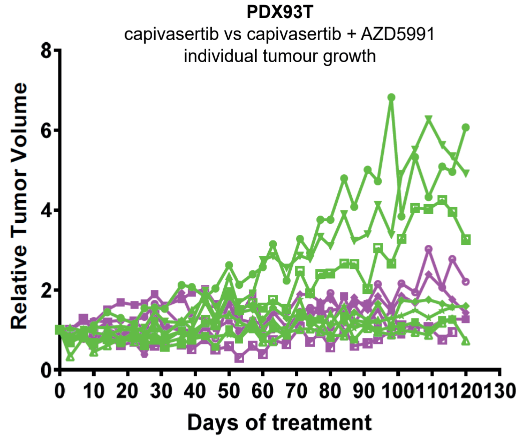

Supplementary Figure 7

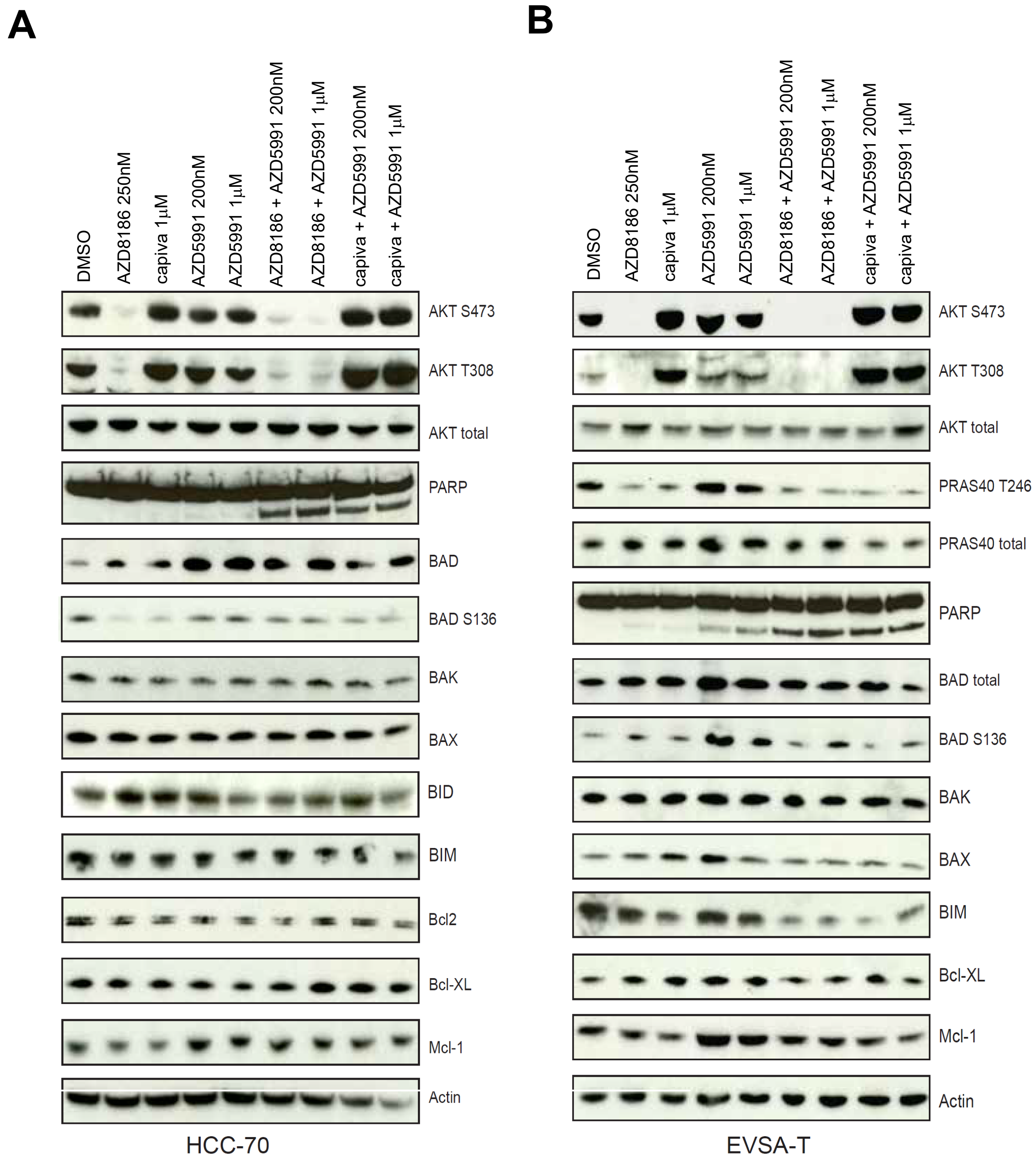

Supplementary Figure 8

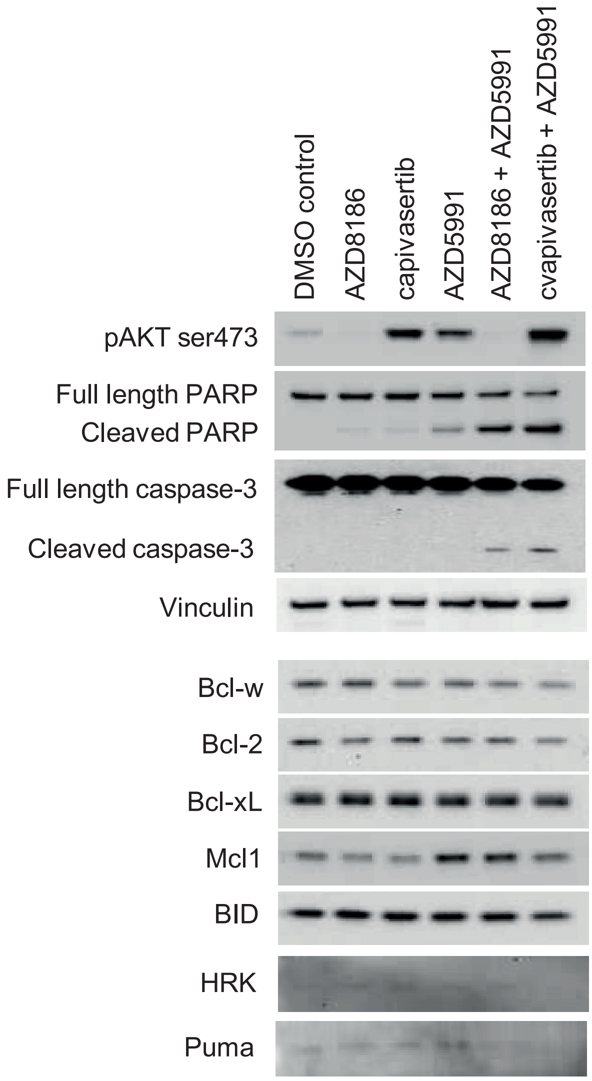

Supplementary Figure 9

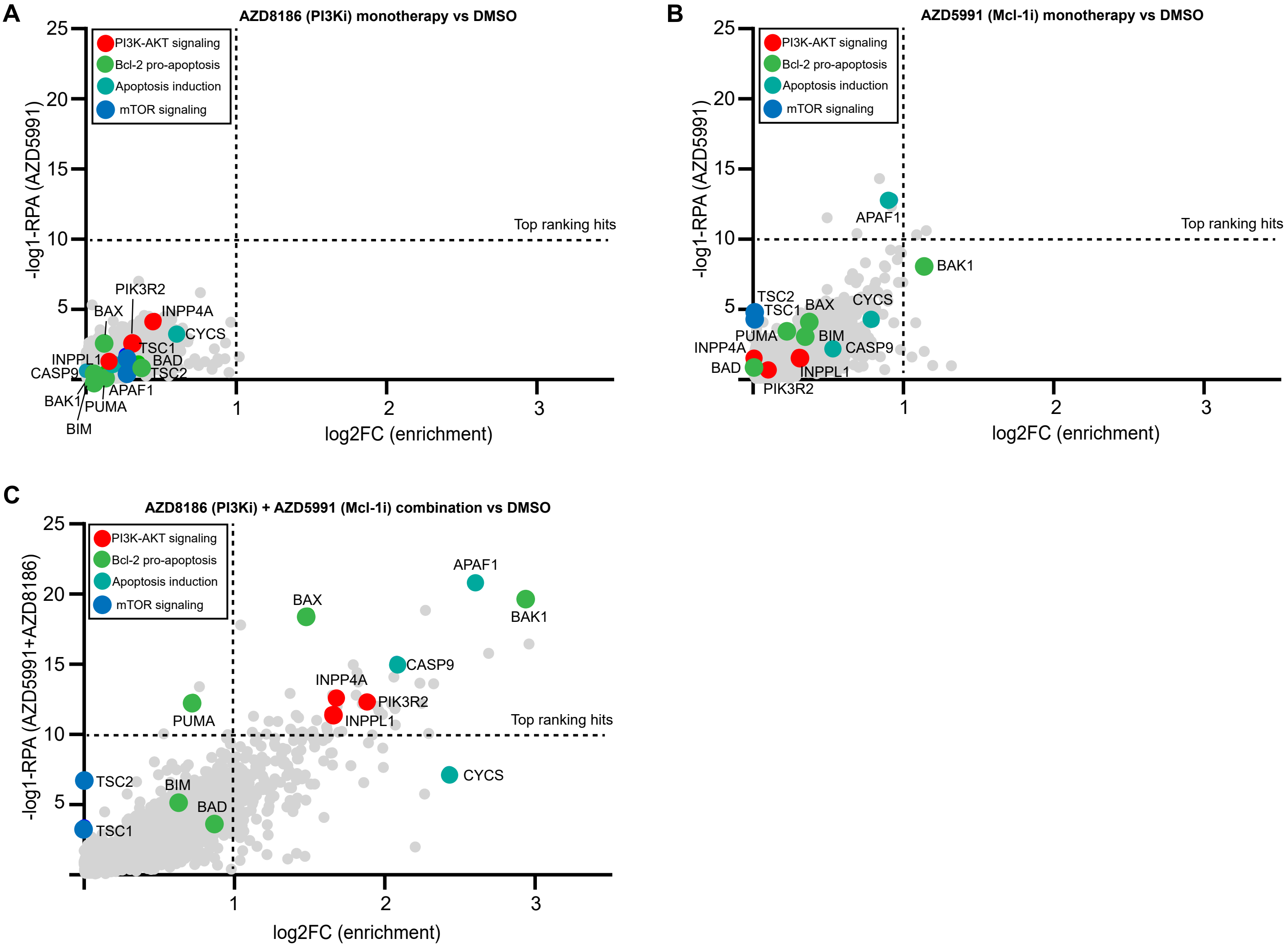

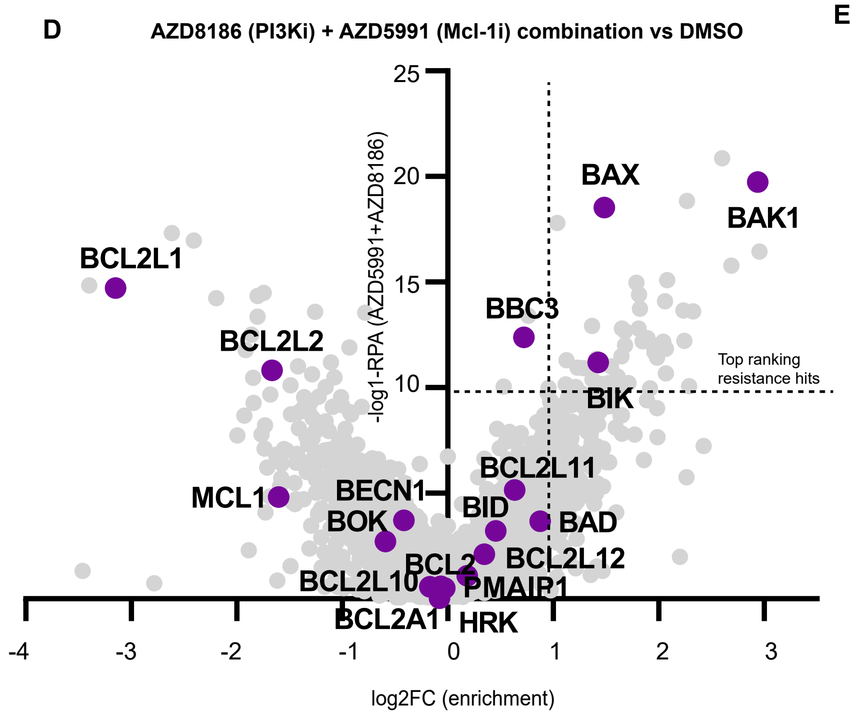

E

Name	Gene name	Bcl2- subfamily
BCL-2	BCL2	Anti-apoptotic
BCL-XL	BCL2L1	Anti-apoptotic
BCL-W	BCL2L2	Anti-apoptotic
BCL-B (BCL2L10)	BCL2L10	Anti-apoptotic
MCL-1L	MCL1	Anti-apoptotic
BFL-1/A1	BCL2A1	Anti-apoptotic
BCL2L12	BCL2L12	Anti-apoptotic
BAX	BAX	Pore-forming executioners
BAK	BAK	Pore-forming executioners
BOK	BOK	Pore-forming executioners
BIM	BCL2L11	BH3-only pro-apoptotic
BID	BID	BH3-only pro-apoptotic
PUMA	BBC3	BH3-only pro-apoptotic
BAD	BAD	BH3-only pro-apoptotic
NOXA	PMAIP1	BH3-only pro-apoptotic
BIK	BIK	BH3-only pro-apoptotic
BMF	BMF	BH3-only pro-apoptotic
HRK	HRK	BH3-only pro-apoptotic
Beclin-1	BECN1	BH3-only pro-apoptotic
